# Perineuronal nets affect memory and learning after synapse withdrawal

**DOI:** 10.1038/s41398-022-02226-z

**Published:** 2022-11-15

**Authors:** Jiri Ruzicka, Marketa Dalecka, Kristyna Safrankova, Diego Peretti, Pavla Jendelova, Jessica C. F. Kwok, James W. Fawcett

**Affiliations:** 1 Institute of Experimental Medicine, CAS, Prague, Czech Republic; 2 Imaging Methods Core Facility, BIOCEV, CAS, Vestec, Czech Republic; 3 UK Dementia Research Institute and Department of Clinical Neurosciences, University of Cambridge, Cambridge, UK; 4 Faculty of Biological Sciences, University of Leeds, Leeds, UK; 5 John van Geest Centre for Brain Repair, University of Cambridge, Cambridge, UK

**Keywords:** Hippocampus, Long-term memory

## Abstract

Perineuronal nets (PNNs) enwrap mature neurons, playing a role in the control of plasticity and synapse dynamics. PNNs have been shown to have effects on memory formation, retention and extinction in a variety of animal models. It has been proposed that the cavities in PNNs, which contain synapses, can act as a memory store and that they remain stable after events that cause synaptic withdrawal such as anoxia or hibernation. We examine this idea by monitoring place memory before and after synaptic withdrawal caused by acute hibernation-like state (HLS). Animals lacking hippocampal PNNs due to enzymatic digestion by chondroitinase ABC or knockout of the PNN component aggrecan were compared with wild type controls. HLS-induced synapse withdrawal caused a memory deficit, but not to the level of untreated naïve animals and not worsened by PNN attenuation. After HLS, only animals lacking PNNs showed memory restoration or relearning. Absence of PNNs affected the restoration of excitatory synapses on PNN-bearing neurons. The results support a role for hippocampal PNNs in learning, but not in long-term memory storage for correction of deficits.

## Introduction

Perineuronal nets (PNNs) are dense extracellular matrix structures surrounding specific neuronal types. In the brain, they are predominantly formed around the fast-spiking parvalbumin positive inhibitory interneurons (PV^+^), as a lattice-like structure on the membrane with regions of uncovered membrane mostly occupied by synapses [1, 2]. They are composed mainly of hyaluronan, chondroitin sulfate proteoglycans (CSPGs), hyaluronan and proteoglycan link proteins (Haplns) and tenascins, with CSPGs providing the main inhibitory properties [2–8]. The PNNs are fully formed at the end of critical period and serve as regulators of plasticity and excitability, and are also neuroprotective [9–11]. The number, composition and anatomy of PNNs can change as a result of various behavioral events during development and ageing through regulation of individual PNN components, and the CSPG sulphation pattern [12–16].

Recently, a role for PNNs and CSPGs in memory has emerged as shown by studies focused on fear [17], object recognition [18–20], associative memory and learning [21], and drug craving and seeking behavior [22–26]. Since memory is encoded in the pattern and strength of synaptic connections, and PNNs regulate synapse dynamics and plasticity, it has been proposed that the lattice-like structure of PNNs might encode long-term memory [27]. An attraction of this idea is that PNNs are relatively stable over time, and remain in place after hibernation and in the period after stroke anoxia before activation of inflammation [28, 29]. Most mechanisms for synaptic maintenance require energy, yet PNNs remain in place after anoxia, and ECM molecules can aggregate and maintain their structure without external energy.

Synaptic withdrawal occurs under various circumstances including stroke, anoxia and hibernation. An unsolved question is how memories can remain stable after prolonged periods of brain anoxia, cooling or hibernation which cause extensive synaptic withdrawal [30–33]. One animal model of short-term hibernation-like state (HLS) involves administration of 5’-AMP combined with cooling to activate the hypothalamic nuclei for induction of torpor [34–37], resulting in 30% withdrawal of synapses in hippocampal CA1, with subsequent recovery [38, 39]. The mechanism for directing these synapses back to appropriate connections is only partially understood.

Could PNNs provide a substrate to stabilize synapses and enable memory retention? The hypothesis that PNNs regulate long term memory makes two predictions. The first is that memories will be reflected in the lattice pattern of PNNs, reflecting the pattern of synaptic connections. The second is that the persistence of memories after hibernation or anoxia will depend on the presence of PNNs. Our study addresses this second prediction.

For this we used the model of synapse withdrawal caused by HLS. The study focused on hippocampal spatial memory, assessed using the Morris water maze (MWM). The involvement of the hippocampus in spatial memory has been shown by multiple techniques, and it contains place cells which are central to spatial memory [40–44] and prominent PNNs mostly around PV^+^ interneurons [45]. We first measured synapse withdrawal and reconnection caused by HLS. This was done in three models that attenuate PNNs, enzymatic digestion of PNNs, local knockout of *Acan* via AAV1-*hSynapsin-Cre* injection in *floxP Acan* mice and global CNS knockout of *Acan* through crossing the *floxP Acan* mice with *nestin cre* drivers. Animals were trained in spatial memory in the MWM, cooled, then memory retention and ability to re-learn were measured in the presence or absence of PNNs. We found that HLS caused synapse withdrawal, particularly on the PNN-bearing PV^+^ inhibitory interneurons. Synapses reconnected after HLS, and the numbers of excitatory regenerated synapses were increased in the absence of PNNs. HLS led to partial loss of the memory and impaired subsequent re-learning. The absence of PNNs did not lead to a greater memory deficit compared to controls. Instead, absence of PNNs accelerated memory recovery and re-learning. We conclude that PNNs in the hippocampus are not required for the retention of spatial memories when the brain suffers an event that causes synaptic withdrawal.

## Results

### Study design and timeline

**Scheme 1:**
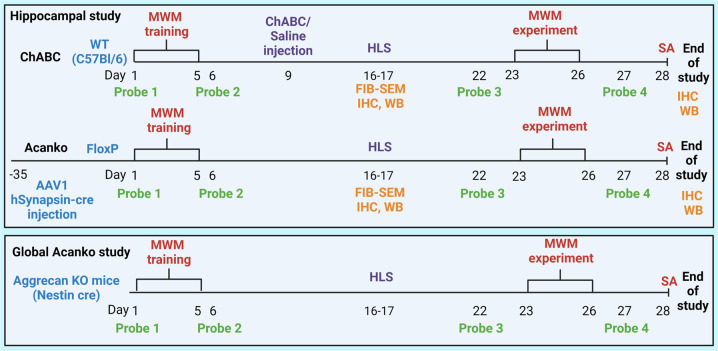
Design of the study.

The aim of the study was to use experimental models of place memory to determine whether the presence of PNNs in the hippocampus affects memory loss after a hibernation-like state (HLS) and subsequent restoration of memory and relearning. Two methods of attenuating PNNs were used in three separate experiments: (1) The enzymatic ChABC experiment; ChABC was injected to 4 hippocampal sites bilaterally to digest PNNs after the initial learning period and before HLS, while controls received a general anesthetic and saline or ChABC injection without HLS (Scheme 1), and in the two transgenic experiments (2) conditional *Acan* knockout (*Acan*KO): *floxP-Acan* mice received injections of AAV1-*hSynapsin-Cre* to both hippocampi 5 weeks before initial training (Scheme 1), eliminating hippocampal PNNs (Fig. S1; Injection sites at the end of the study, Fig. S2; CA1 and PV^+^ neuron detail of WFA, PV^+^ signal) and (3) global CNS *Acan* knockout animals were bred by crossing the *floxP-Acan* animals with *nestin-cre* animals, eliminating PNNs throughout the nervous system. All animals were initially tested in the MWM probe test to establish the performance of naïve animals, then they were trained for 5 days to learn the location of a submerged platform in the MWM and to measure initial learning. In the enzymatic experiment, ChABC or saline was injected on day 8–9, (i.e. 3 days after the training period) in order to ensure that PNNs were fully digested before their gradual replenishment over ~3 weeks [45, 46] (Figs. S1 and S2). In the hippocampal *Acan*KO experiment AAV1*-hSynapsin-Cre* was injected 5 weeks before initial learning in order to give time for the existing Acan to disperse (Figs. S1 and S2). In global CNS *Acan*KO animals, Acan was absent from birth and this was checked at the end of a study (Fig. S11). In all groups, half of the animals were placed in HLS over 1 day to induce synaptic withdrawal with the other half serving as controls. After 1 week to allow sufficient recovery, on day 22 a MWM probe test with platform removal was done to analyze memory retention after HLS. The MWM was performed and repeated daily for 5 days in order to measure relearning. A probe test in which the submerged platform was removed was performed again at the end of this week on day 27. As a general test of hippocampal function, spontaneous alternation in the Y-maze was performed on day 28 after which the animals were perfused for histology.

### HLS temporarily reduces synapse numbers

**Fig. 1:**
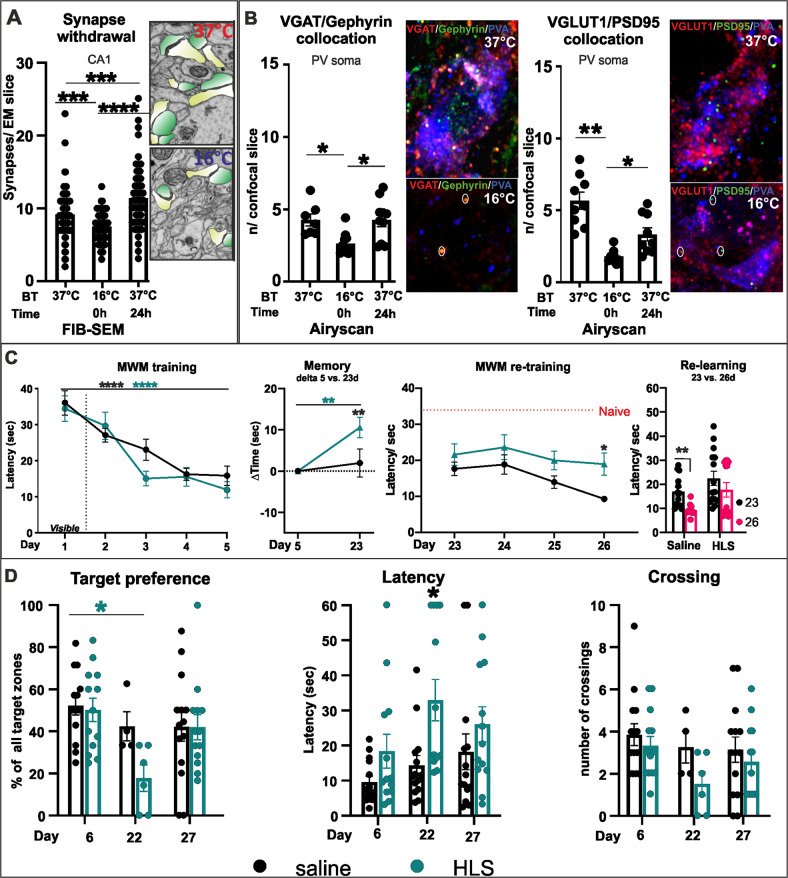
A, B The synapse withdrawal effect of HLS in CA1. The samples were taken before HLS, immediately after 45 min cooling to 16–18 °C and 24 h later after passive warming to 37 °C. **A** Total synapse number (representative image showing impact of HLS; pre and post synaptic site marked by yellow and green, respectively). **B** Counts of synapses on PV^+^ inhibitory interneuron soma. Pre- and postsynaptic components co-localized on PV^+^ signal were evaluated on single neuron confocal Z-stack series (under Airyscan super-resolution mode) and newly appearing co-locations per slice were counted. There was withdrawal during HLS followed by complete recovery in inhibitory synapses (vGAT/Gephyrin), but only partial recovery of excitatory synapses (vGLUT/PSD95). **C** Memory measured on the Morris water maze (MWM) over the entire experiment, comparing animals subjected to HLS and controls. **C** (left) Before the HLS procedure WT animals trained for 5 days displaying normal learning curves with no difference between the groups (TW-RM-ANOVA *F* (1,24) = 0.9464 *p* = 0.34). **C** (middle) HLS caused a memory deficit between days 5 and 23, although not to the level of Naïve untrained animals (TW-RM-ANOVA *F* (1,24) = 4.338 *p* = 0.0481). **C** (right) After the HLS period, starting at day 23, animals returned to the MWM for daily training. WT animals relearned, but HLS animals did not (TW-RM-ANOVA *F* (1,24) = 5.043 *p* = 0.0342). **D** At days 6 and 22 (before and after the HLS period) probe tests without the platform were performed. HLS animals showed significant loss of target preference (time spent in target zone), increased latency to reach the target (Latency, TW-ANOVA *F* (3,48) = 4.269 *p* = 0.0094) and decreased frequency of crossing of target area. Comparing the beginning and end of the relearning period, HLS animals recovered target preference, but only partially recovered latency and crossing. Significance *p < 0.05, **p < 0.01, ***p < 0.001. Statistics Table S1. (**A** *n* = 66 sections per time point; **B** *n* = 2–3 neurons per group/time point (av. ratio from Z-stack planes images, at least 20 images per neuron); **C**, **D** saline *n* = 13 HLS *n* = 14 animals).

The first step was to confirm that the induction of HLS leads to temporary synaptic withdrawal in the hippocampus as previously shown and to examine the effects on memory. Animals were subjected to HLS, cooling to a core temperature of 16–18 °C, maintained for 45 min then re-warmed over 24 h, as in the previous study [39]. The effect of the HLS on synapse numbers in the CA1 dendritic tree area was measured. Focused ion beam- scanning electron microscopy (FIB-SEM) was used to assess overall synapse numbers. Sections of CA1 were taken from animals before HLS (37 °C), immediately after HLS (16–18 °C) and 24 h after HLS by which time the body temperature had recovered to 37 °C. In the sections, all synapses in selected areas were counted, defined by the co-location of a vesicle-containing presynaptic element and a thick or thin postsynaptic density on a dendritic figure. We observed that numbers of synapses decreased at the end of HLS, followed by restoration of synapse numbers at 24 h to a slightly higher number (Fig. 1A). There was a 20% (±2.64) reduction of synapse numbers after HLS, then recovery with ~20% (±5.38) overshoot. These results reproduce the original model published by [39].

PNNs in the brain predominantly surround PV^+^ inhibitory interneurons which are implicated in the control of memory in the cortex and hippocampus [8, 47]. We performed analysis of individual pre and postsynaptic markers for excitatory and inhibitory synapses located on PV^+^ neurons, using colocalization of vGlut and PSD95 as a marker for excitatory synapses, and that of presynaptic vGAT and postsynaptic gephyrin for inhibitory synapses. The number of synaptic compartments on single optical sections of PV^+^ neuronal bodies was counted (Fig. 1B). Immediately after HLS (16–18 °C) a large reduction in excitatory and inhibitory synapse number was detected (Fig. 1B). After the 24 h passive rewarming phase (37 °C), excitatory and inhibitory synapses recovered to values that were not significantly different to pre-HLS. In detail, there was a 38% (±5.71) reduction of inhibitory and 68.5% (±2.76) reduction of excitatory synapses after HLS. After 24 h recovery, inhibitory synapse numbers returned to normal, but excitatory synapses returned to 58.6% (±7.68) of pre-HLS levels. We also counted the numbers of orphan terminals, those without the co-localization with postsynaptic markers, of bassoon and vGAT (Fig. S3). There was a 78% (±1.87) reduction of vGAT and 42% (±8) reduction of bassoon positive terminals after HLS. After 24 h rewarming, the number of vGAT terminals was 65% (±5.19) of the original level while the bassoon terminals fully recovered at 95% (±10.21). HLS also led to reductions in PSD95, SNAP25, vGLUT1 and vGAT measured by western blots (Figs. S5, S6 and S12).

#### HLS affects MWM memory

In order to study the effects of cooling and PNN manipulation on long-term memory, we tested place memory using the MWM. In this task animals learn the position of a refuge platform placed under the opaque water surface. This task is widely used to reveal spatial memory dependent on the hippocampus, and memory is adversely affected by various hippocampal interventions [40, 42]. After learning the position of the platform, the memory is usually stable for 4–5 weeks in normal animals.

The effect of HLS on memory in normal animals was assessed. Animals were trained to remember the position of the refuge platform by daily training for 5 consecutive days in the MWM, during which the latency time needed to find the target (first crossing of the platform boundary, with at least 0.5 s spent at the target platform) was measured. Mice were then subjected to HLS in which their body temperature was dropped to 16–18 °C for 45 min, causing synaptic withdrawal as described above. Animals needed 1 week to recover from this procedure, after which their memory for the position of the platform in the MWM was tested again. For five further days animals were tested in the MWM and their ability to re-learn the refuge position was measured (Fig. 1C). Probe tests in the absence of the platform were performed on day 6 before HLS, 7 days after HLS (day 22) and on the final day (day 27; timeline Scheme 1 and Fig. 1D). During the 5 training days, animals showed a progressive decrease in latency for finding the target. Comparing memory before and after HLS, animals showed a significant increase in latency, although not to the naïve pre-training level (Fig. 1C). Memory loss was confirmed by comparing pre- and post- HLS probe tests, with changes in target preference, latency and target boundary crossing (Fig. 1D). In the 5 relearning days after the HLS period, HLS animals showed no significant relearning in the latency and probe tests while animals not subject to HLS continued to learn with shortening latency (Fig. 1C, D). Overall, these results show that animals subjected to HLS demonstrated a period of synaptic withdrawal including withdrawal of synapses on PV^+^ interneurons, and a partial loss of MWM memory. During the relearning period HLS animals failed to relearn, unlike non-cooled animals which continued to learn. Together these results show that HLS causes synaptic withdrawal followed by regeneration, and a partial memory deficit followed by impaired relearning.

### Removal of PNNs affects recovery of synapse numbers after HLS

**Fig. 2:**
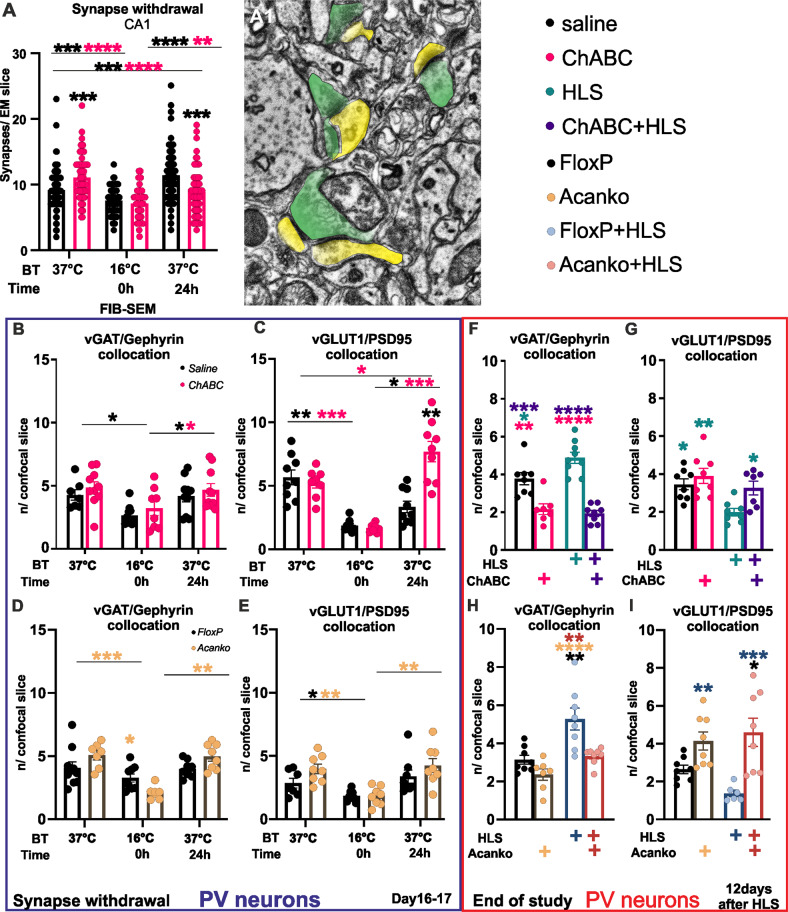
Effect of ChABC PNN digestion 1 week before HLS on synaptic changes. **A** Focused ion beam-scanning electron microscopy (FIB-SEM) of synapses in the CA1 dendritic tree area before, immediately and 24 h after HLS pre-synapses are green, dendrites yellow. At the initial euthermic condition, the ChABC treated CA1 area showed an increased number of synapses. Immediately after HLS in control and ChABC groups a drop-in number of synapses was observed. After 24 h rewarming period both groups recovered synapses to the euthermic level (Illustration **A**1, detail **A**2). **B**–**E** Effects of HLS on excitatory and inhibitory synapses on parvalbumin^+^ interneurons. Collocation of presynaptic terminals (vGAT (**B**, **D**)/vGLUT (**C**, **E**)) and postsynaptic densities (Gephyrin (**B**, **D**)/PSD95 (**C**, **E**)) were evaluated on single neuron confocal Z-stack series (under Airyscan super-resolution mode) and newly appearing co-locations per slice were counted. Both excitatory and inhibitory synapses decreased after HLS with recovery at 24 h. The impact of the PNN integrity was significant, with a robust increase of excitatory synapses on PV^+^ neurones in comparison with intact PNNs. **F**–**I** Long-term impact of integrity of PNNs was observed at the end of behavioral study. HLS has shown increase in inhibitory synapses (**F**, **H**) and decrease in excitatory synapses on parvalbumin^+^ interneurons. This long-term impact of PNN integrity was significant, decreasing the inhibitory synapses and increasing excitatory ones. Illustrative images of measured samples are shown. Significance *p < 0.05, **p < 0.01, ***p < 0.001. Statistics Table S2. (**A** *n* = 66 sections per group/time point; **B**–**I** *n* = 2–3 neurons per group/time point (av. ratio from Z-stack planes images)). Statistical color coding: Between different time points the asterisk color coding indicates in which group the statistical significance was found. Within the single time points, the asterisk color coding indicates to which group the statistically significant difference was detected.

The effect of PNNs on synapse numbers after recovery from HLS was examined. The ChABC group received injections to CA1 to digest the PNNs 1 week before HLS. Digested PNNs are not fully replenished for over 3 weeks [46]. Overall synapse number was measured by FIB-SEM. Before HLS, there were more active synapses per section in the ChABC pre-treated group than in saline controls (Fig. 2A). At the end of HLS, both ChABC and control saline-treated groups had decreased numbers of synapses, followed by restoration of synapse numbers at 24 h. In the saline-treated group the synapse number was slightly higher than in the ChABC group.

**Fig. 3:**
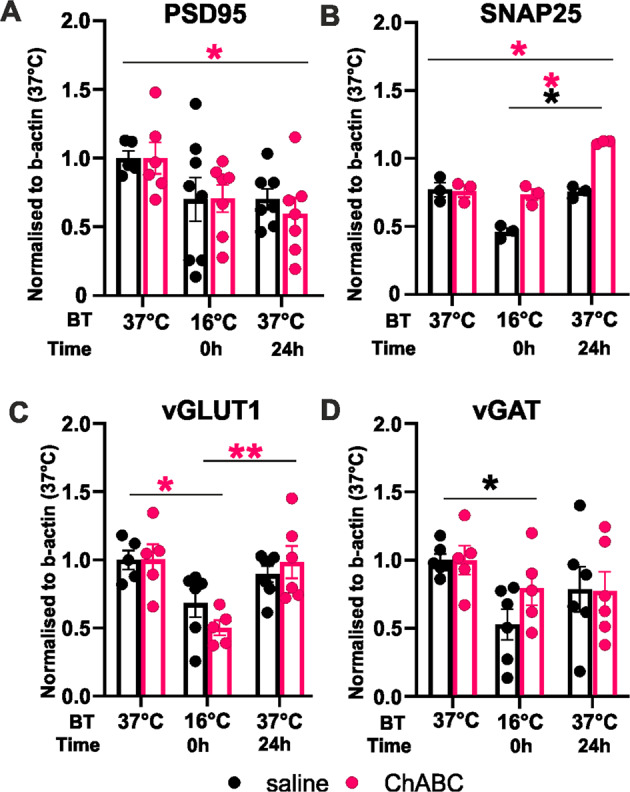
Western blot analysis of pre- and postsynaptic markers from hippocampus lysate before, at the end of HLS and after 24 h recovery. The level of the presynaptic markers SNAP25, vGLUT1, vGAT dropped after HLS, then recovered after 24 h. The postsynaptic marker PSD95 showed no change, the recovery phase was not detected. ChABC treatment increased presynaptic SNAP25 particularly after rewarming (SNAP25, TW-ANOVA, Group *F* (1, 4) = 32.85 *p* = 0.0046). Western blot illustrative images in the supplements. Significance *p < 0.05, **p < 0.01, ***p < 0.001 Statistics, Stat Table 3. Illustrative blot images in supplement. (**A**, **C**, **D** *n* = 5–7 animals per group/time point; **B** *n* = 3 animals per group/time point). Statistical color coding: Between different time points the asterisk color coding indicates in which group the statistical significance was found. Within the single time points, the asterisk color coding indicates to which group the statistically significant difference was detected.

Synapse numbers on PV^+^ interneurons were also affected by ChABC digestion. Just after HLS (16–18 °C) a large reduction in excitatory (vGlut and PSD95) and inhibitory (vGAT and gephyrin) synapses was detected in ChABC and control groups (Fig. 2B, C). After the 24 h rewarming phase, excitatory synapse numbers returned to a level comparable to the saline control group, but in ChABC- treated animals the number of excitatory synapses was more than double to that of the post-HLS control control group and 50% greater than pre-HLS levels (Fig. 2C). A similar increase was seen in the number of bassoon terminals (Fig. S3). The number of inhibitory synapses decreased after cooling and recovered to pre-HLS values in 24 h. In hippocampal *Acan*KO animals, there was a decrease in the numbers of excitatory and inhibitory synapses on HLS with recovery at 24 h with no differences between groups (Fig. 2D, E). Synaptic proteins PSD95, SNAP25, vGLUT1 and vGAT were measured by western blot. There is a general reduction of synaptic markers with HLS, with SNAP25 and VGLUT1 recovering to pre-HLS values at 24 h (Fig. 3).

Overall, the results above confirm that subjecting animals to a hibernation-like state led to a temporary withdrawal of synapses in the hippocampus. Both excitatory and inhibitory synapses were withdrawn more markedly from PV^+^ interneurons. After rewarming, synapse numbers were partly or completely restored. Injection of ChABC had a marked effect on synapses in PV^+^ interneurons, with an increase in excitatory synapses compared to controls. Biochemical assay of synaptic proteins showed a similar pattern, with a decrease after HLS which was largely restored on rewarming.

### PNN digestion or attenuation does not increase memory loss but promotes relearning

The results described above demonstrate that induction of HLS in mice led to a partial loss of place memory after recovery, followed by a period in which animals did not relearn. We asked whether the presence of PNNs in the hippocampus would affect these processes. As above, three separate experiments were run, using different methods of PNN manipulation. (1) digestion of PNNs with chondroitinase ABC (ChABC) (ChABC experiment), (2) local attenuation of PNNs in *Acan*-*floxP* animals through injection of AAV1-*hSynapsin-Cre* bilaterally into the hippocampus (Acan experiment), (3) global *Acan*KO in the CNS through crossing *Acan-floxP* mice with *nestin-cre* mice (Global Acan experiment) (timeline Scheme 1 and Figs. S1, S2 and S11). In all these experiments removal of PNNs preceded HLS, but ChABC was injected after the initial learning phase just before HLS in order to ensure that PNNs were depleted during the HLS and relearning period [46], while AAV1-*hSynapsin-Cre* was injected prior to initial learning and the global knockouts lacked CNS Acan from embryos. Thus, for the ChABC experiment all animals were untreated WT during the initial training period, but the aggrecan knockout animals had attenuated hippocampal PNNs throughout.

**Fig. 4:**
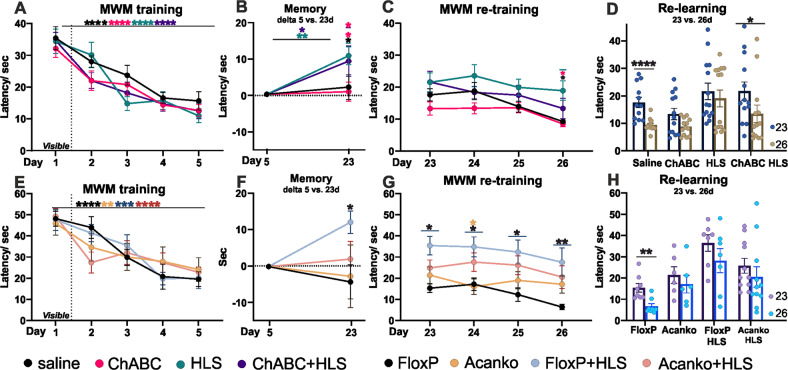
Morris water maze; effects of PNN removal on memory before and after HLS. ChABC experiment (**A**–**D**) and *Acan*KO experiment (**E**–**H**). AAV-*Cre* to induce *Acan*KO was injected before initial training, ChABC after initial training. All, WT, *floxP* and *Acan*KO mice showed normal learning (**A** (day 1 vs. day 5; TW -RM-ANOVA, Time: *F*(4,192) = 45.68, *p* < 0.0001, Group *F*(3,48) = 0.927 *p* = 0.44, **E** (TW -RM-ANOVA, Time: *F*(4,104) = 26, *p* < 0.0001, Group *F*(3,26) = 0.202 *p* = 0.99)). The training week was followed by ChABC injection (**A**–**D**) followed by HLS, then memory testing and training resumed on day 23. Hibernated animals showed a partial loss of memory, but not to the level of naïve animals (**B**). This loss had already recovered by day 23 in *Acan*KO ^+^ HLS mice (**F**). During the relearning phase (**C**, **D**, **G**, **H**), animals in the HLS (**C**, **G** detail analysis **D**, **H**) group did not show significant re-learning. However, animals treated with ChABC and HLS were fast relearners (**D**, day 23 vs. 26, saline *t* = 4.751 *p* = 0.002, ChABC HLS, *t* = 2.885 *p* = 0.041). In the *Acan*KO + HLS animals’ memory was not lost during the HLS period, suggesting rapid relearning during this period (**F**). Generally, FloxP HLS animals showed worsened performance in re-training week in comparison to floxP control, but no statistical difference between *Acan*KO and *Acan*KO HLS was found (re-training, TW-RM-ANOVA, Group: *F*(3, 26) = 6.155 *p* = 0.0026, FloxP vs. FloxP HLS *q* = 5.737 *p* = 0.0021, *Acan*KO vs. *Acan*KO HLS *q* = 1.943, *p* = 0.526). Significance *p < 0.05, **p < 0.01, ***p < 0.001. Statistics Table S6. (this Figure saline *n* = 13, HLS *n* = 14, ChABC *n* = 13, ChABC HLS *n* = 14, FloxP *n* = 8, FloxP HLS *n* = 6, *Acan*KO *n* = 6, *Acan*KO HLS *n* = 10 animals).

During the initial learning period the time taken to find the platform in the WT animals in the ChABC experiment decreased from 33 s to 14 s. In the hippocampal and global Acan experiments, there was no difference between *Acan*KO and saline control animals, the time taken to find the platform decreased from 50 s to 24 s (Figs. 4A, E and S9).

#### Hibernation and PNN removal affect MWM memory

We asked whether PNN attenuation affects the memory loss that occurs after HLS.

##### ChABC experiment

**Fig. 5:**
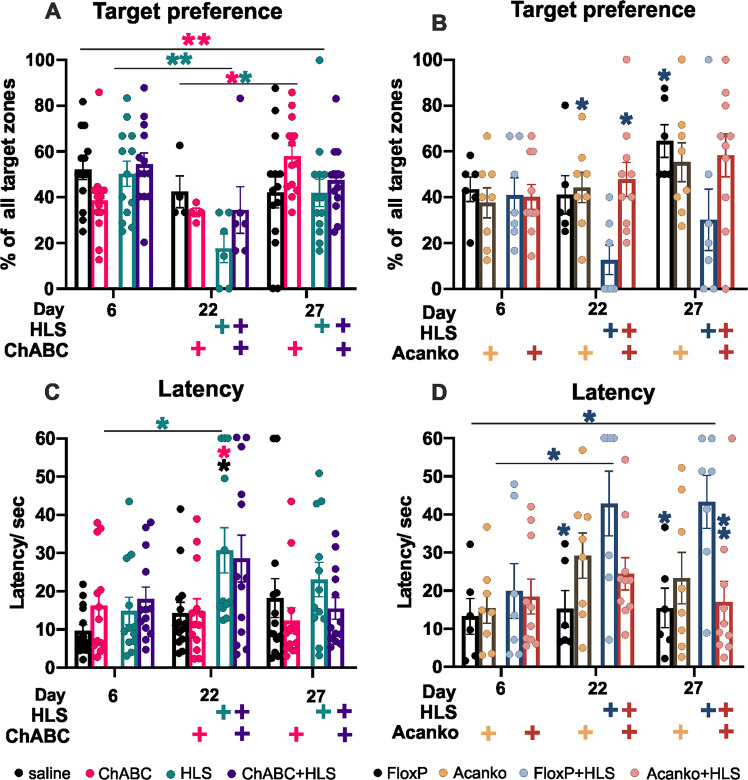
Probe test with target platform removed, comparing the 6th and 22nd and 27th days, assaying memory at the end of pre-training, after HLS and after relearning. Target preference and latency are shown here, other measures in Fig. S5. There are no significant differences between groups after the first training session, but HLS causes loss of target preference and increased latency in all but the *Acan*KO group **A**–**D**. (TW-RM-ANOVA, Preference, Group *F*(3,27) = 5.282 *p* = 0.0054, FloxP HLS vs. FloxP *q* = 4.651 *p* = 0.014, FloxP HLS vs. *Acan*KO HLS *q* = 5.028 *p* = 0.0073, *Acan*KO vs. *Acan*KO HLS *q* = 0.7804 *p* = 0.945; Latency, Group *F*(3, 48) = 4.269 *p* = 0.0094, HLS vs. saline *q* = 4.130 *p* = 0.0264, ChABC HLS vs. ChABC *q* = 2.890 *p* = 0.187; Latency, group, *F*(3, 27) = 5.817 *p* = 0.0033, FloxP HLS vs. FloxP *q* = 5.526 *p* = 0.003, FloxP HLS vs. *Acan*KO HLS *q* = 4.651 *p* = 0.014). All groups show some relearning (statistics Tables S7 and S8 in attachments). Significance *p < 0.05, **p < 0.01, ***p < 0.001. (Fig. 4 saline *n* = 13, HLS *n* = 14, ChABC *n* = 13, ChABC HLS *n* = 14, FloxP *n* = 8, FloxP HLS *n* = 6, *Acan*KO *n* = 6, *Acan*KO HLS *n* = 10 animals).

Half of the trained WT animals received ChABC injections to the hippocampus after the training period, the other half were injected with saline. The ChABC treatment caused loss of *Wisteria floribunda* agglutinin (WFA)-stained PNNs in the hippocampus, usually extending into the neighboring cortex (Figs. S1 and S2C). After recovery from the surgery (1 week) half the ChABC and saline mice were placed in HLS, non-HLS animals serving as controls. Animals needed 1 week to recover from this procedure, after which their memory of the position of the platform in the MWM was tested again with a probe test in the absence of the platform and then with daily MWM testing. The two groups of animals that underwent HLS (HLS-ChABC and HLS-saline) showed a partial loss of place memory at the first latency test after HLS, with no difference between ChABC and saline treated groups (Fig. 4B). Their latency time to find a target had increased, but not to the time score of naïve animals. In the target preference probe test the post HLS score of the ChABC group suggested some recovery during the week after HLS before the first MWM tests (Fig. 5A) (see below).

##### Focal and global Acan knockout experiments

One week after the last training session, half of the *Acan*KO and *floxP* animals underwent HLS the others serving as controls, then their memory in the MWM was tested 1 week later. In the hippocampal focal *Acan*KO HLS experiment, HLS control animals showed a memory deficit, although not to the level of naïve animals. However, in HLS focal *Acan*KO mice the latency at 1 week after HLS was at the same level as on the last training day (Fig. 4F). The probe tests all showed a similar result with no significant memory loss between the end of training and first tests after HLS, while the HLS controls showed a tendency to memory loss (Fig. 5). Our interpretation of this result is that the animals lacking PNNs in the hippocampus had rapidly recovered memory during the week after HLS and before the first MWM tests. The logic is that all the HLS animals including those lacking PNNs showed the same amount of synapse withdrawal after HLS (Figs. 2 and S3) which causes a memory deficit. These results therefore suggest that in the animals lacking PNNs in the hippocampus there had been rapid recovery of memory in the week following HLS before testing, while controls with PNNs did not show memory recovery during this period. The global AcanKO and HLS control animals showed a partial and similar loss of memory between the end of training and the first tests 1 week after HLS with no evidence of rapid recovery during the post-HLS period (Fig. S9B) The probe tests for this experiment showed a similar result (Fig. S10). Attenuation of PNNs per se does not therefore prevent memory loss after HLS.

### Absence of PNNs enables re-learning after HLS memory deficit

The re-learning phase started with the MWM tests 1 week after HLS. Daily testing was performed for a further 4 days to measure relearning at which point there was a further probe test (Scheme 1 timeline). In the ChABC experiment, the two groups that had not been subjected to HLS showed a further progressive shortening in their latency time to reach the target (Fig. 4C). The two groups (control and ChABC) that received HLS started the relearning period with a memory deficit compared to before HLS. Of these, the saline-treated HLS controls showed no significant re-learning (Fig. 4D). However, the ChABC pre-treated animals were able to re-learn the task (Fig. 4D). Effects of ChABC on PNNs last for at least 3 weeks due to persistence of the enzyme and slow PNN replacement [46], so the relearning enhancement occurs during PNN attenuation.

In the hippocampal focal *Acan*KO experiment, the control HLS *floxP* controls started the re-learning period with a memory deficit after which they showed no significant relearning (Fig. 4F, H). The HLS focal *Acan*KO group started this period with no deficit, suggesting that they had already compensated for the effect of HLS before the first post-HLS test, and then did not show further relearning. In the probe tests at the end of the re-learning week, the *floxP* + HLS group performed consistently worse than the other groups and did not relearn (Figs. 5 and S7).

In the global *Acan*KO experiment both *Acan*KO and control groups had a memory deficit after HLS, and therefore started relearning with a deficit. This result also shows that absence of Acan does not prevent memory loss after HLS. The HLS global *Acan*KO group showed significant relearning, and the HLS control group showed a non-significant trend (Figs. S9 and S10).

For all the MWM experiments, we measured swim speeds. There was no difference between the various experimental groups and controls.

Together, these results show that HLS produces a memory deficit although not a complete loss of memory to the level of naïve animals. Attenuation of PNNs did not increase this memory loss. Control HLS animals with intact PNNs did not show significant relearning following HLS, whereas control non-HLS animals continued to learn during the relearning period. However, the three HLS groups with attenuated PNNs showed restoration of memory during or before the relearning period. The timing of restoration of memory varied between groups. The hippocampal focal *Acan*KO animals recovered rapidly during the 1 week between HLS and the first MWM tests, the ChABC animals recovered during the relearning period with one task recovering faster during the week after HLS, and the global *Acan*KOs showed no recovery during the week after HLS but then marked relearning during the relearning period.

An alternative test of ventral hippocampal memory function is spontaneous alternation, in which animals are placed in a Y-maze and their spontaneous entry in the arms is recorded. A hippocampal deficit can cause animals to re-enter the same arm rather than alternating between arms. This test was performed at the end of the re-learning week. We did not find any difference between any of the experimental groups (ChABC, *Acan*KO or global *Acan*KO experiment, Fig. S8). This indicates that by 1 week after hibernation hippocampal function is normal.

## Discussion

PNNs form a lattice around PV^+^ interneurons and some other neurons. Because PNNs contain inhibitory CSPGs, it is probable that regenerated synapses will tend to reconnect to the areas of naked neuronal membrane that they previously occupied rather than penetrating the PNNs. This led to the idea that PNNs might specify memory by constraining the positions of synapses. An unsolved question is how memories can remain stable after prolonged periods of brain anoxia, cooling or hibernation which cause extensive synaptic withdrawal [30–33], leading to the hypothesis that PNNs might be the substrate for stable memories following conditions that lead to synapse withdrawal [27]. Our experiments were designed to test this hypothesis for hippocampal-based place memory. The first step was to confirm that HLS leads to synapse withdrawal followed by reconnection in our HLS model, as previously shown [39]. HLS led to synaptic withdrawal in the hippocampus, particularly on PV^+^ inhibitory interneurons, followed by restoration to near the original level. Removal of PNNs with ChABC led to increased numbers of excitatory synapses on PV^+^ interneurons (the location of the majority of the PNNs) following the regeneration phase 24 h after hibernation. We then asked whether this HLS-induced synaptic withdrawal would lead to memory loss. We recorded partial loss of MWM place memory after HLS, although not to the level of naïve untrained animals. Step 3 was to ask whether removal of PNNs before HLS would lead to a greater loss of MWM place memory than in animals with intact PNNs. This did not happen. Digestion or deletion of ECM/PNNs did not increase the memory loss due to HLS, suggesting that PNNs on hippocampal neurons are not a long-term store for positional memory.

Many previous studies have suggested that PNN depletion can enhance memory acquisition and/or persistence (see below). We therefore asked whether PNN removal affects learning of the platform position on the MWM, and whether relearning after the memory deficit caused by HLS is affected. Initial learning was not affected by PNN removal, in agreement with previous work in which PNNs were attenuated by tenascin-R knockout [48]. Following the HLS period, animals were assessed for their ability to relearn, receiving daily MWM testing followed at the end by another probe test. Control animals that had not received HLS continued to learn during this period. HLS animals with normal PNNs did not show significant relearning indicating that HLS causes a lasting learning deficit. However, HLS animals lacking PNNs due to ChABC, hippocampal or global AcanKO all recovered or relearned. The hippocampal focal HLS AcanKO animals recovered normal memory fastest before the first post-HLS probe test then did not improve thereafter. The HLS ChABC group did not recover during the HLS period except in target preference but then relearned during the relearning period. The global HLS *Acan*KO group showed no recovery during the HLS period, but then relearned robustly during the relearning period. These results suggest that HLS causes a learning deficit which is compensated by the increased plasticity resulting from PNN removal. These relearning results match with previous work in which lack of PNNs has had a positive effect on memory acquisition and retention [18, 19, 49].

While the hippocampus has been accepted for many years as the central site for place memory, other sites input to the hippocampus and store place information. The entorhinal cortex contains grid cells which connect to hippocampal place neurons, and the cingulate cortex and some subcortical structures are also implicated [50–53]. It is probable that cortical positional backup memory was responsible for the rapid recovery of memory in the hippocampal focal *Acan*KO group during the period between HLS and re-exposure to the MWM. Why did we not see a similar rapid recovery in the ChABC and global *Acan*KO groups? In both these groups PNNs were completely or partially disrupted in the cortical regions lateral to the hippocampus, which is where cortical place memory is found. It is probable that the backup memory store outside the hippocampus does not function efficiently in the absence of PNNS, probably due to the instability in cortical positional networks seen after PNN removal [53].

Several previous PNN modification experiments have focused on the hippocampus. Animals lacking tenascin-R have attenuated PNNs, and these animals showed normal learning rates but faster adjustment to new platform positions [48]. On the contrary, animals with increased numbers and thickness of PNNs showed slow place learning, normalized after ChABC digestion in the hippocampus [54]. PNN digestion also affects hippocampal physiology; digestion of PNNs in CA1 restored long term depolarization (LTD) that is lost in aged mice and enhanced excitability [55], and both ChABC and tenascin-R knockout decrease CA1 long term potentiation (LTP) [56]. In CA2 LTP and excitability were increased by digestion of PNNs, which in this region surround pyramidal cells [57, 58]. PNN removal affects memory and plasticity largely through effects on PV^+^ interneurons, which affect network activity through modulation of GABA-mediated plasticity [45, 47, 59].

The three ways in which we depleted PNNs have different targets. ChABC digests all CS-GAGs, affecting the condensed PNNs visible with WFA staining, but also all the CS-GAGs in the diffuse matrix that surrounds all synapses. Aggrecan is localized to PNNs, so knockout affects PNNs specifically, for which aggrecan is a necessary structural component [49, 60]. *Acan*KO affected relearning to the same extent as ChABC, suggesting that PNNs are involved rather than the diffuse CNS ECM. The short-term HLS model used in this study was developed to study the effects of neurodegenerative conditions on synapse regeneration [39], and reproduces the synaptic withdrawal observed in hibernating together with a torpid state with altered respiration, heart rate and metabolism rates similar to hibernating mammals [30, 34–36]. The same withdrawal and reconnection of synapses was observed in the current study (Fig. 1A).

There have been various studies of the effect of PNN manipulation on memory acquisition and retention, but none in the context of acute synaptic withdrawal. The first study used fear conditioning as the model, with digestion of PNNs in the amygdala. Here, PNNs in adults make memories resistant to erasure by extinction training, while PNN digestion re-enabled the immature situation where fear memory can be erased, a form of reverse learning [17]. Digestion of PNNs in the auditory system also led to increased learning flexibility [61]. Digestion of PNNs in secondary visual cortex disrupted fear memory recall, probably by destabilizing network synchronization [62], and removal of hippocampal PNNs disrupted contextual and trace fear memory [63]. PNNs in the hippocampus and cingulate cortex have been linked to fear memory consolidation, and PNN numbers actually increased in the hippocampus after fear conditioning, and increasing PNN numbers by transduction with Hapln-1 enhanced memory [64]. Several studies have used novel object recognition (NOR), a task dependent on perirhinal cortex, as the memory model. Here, digestion of PNNs with ChABC or attenuation of PNNs through *Ctrl1* knockout, led to prolongation of object memory coupled with increased LTD in the perirhinal cortex [19]. Similarly, ChABC or treatment with an antibody recognizing the inhibitory 4-sulfated CSPGs found in PNNs, was able to restore normal NOR memory in an Alzheimer models (P301S tauopathy and the APP/PS1 amyloid model) [18, 65, 66]. PNN digestion also has effects on addiction memory [67]. PNNs also respond to neurodegenerative diseases, and are associated with psychiatric conditions including schizophrenia, autism and depression [67–71].

Overall, our experiments confirm that HLS leads to a temporary global withdrawal of synapses in the CA1 region of the hippocampus followed by regeneration. Synaptic withdrawal was particularly marked on PV^+^ interneurons most of which bear PNNs. Disruption of PNNs in the hippocampus did not affect initial MWM learning, and did not increase memory loss immediately after HLS, suggesting that hippocampal PNNs are not responsible for memory retention during events that cause synaptic withdrawal. However, removal of PNNs had a positive effect on recovery of memory and relearning in the days after HLS.

## Methods

### Animals

Experiments using enzymatic digestion (chondroitinase ABC) of PNNs were conducted on males with C57Bl/6J background (age = 14 weeks, weight = 27 ± 2 g, *n* = 100). Animals for behavioral experiments were separated in following four groups; saline (*n* = 13), hibernated like state (HLS, *n* = 14), ChABC (*n* = 13), ChABC + HLS (*n* = 14), from which, additionally, IHC and WB analysis (Both *n* = 4/group) was performed. Animals for FIB-SEM microscopy, acute IHC or acute WB (*n* = 3–4/group/time point) were used to observe the dynamic changes in synaptosome.

Experiments with local *Acan*KO via AAV1-*hSynapsin-Cre* is achieved with stereotaxic hippocampal injections on *Acan* GT3^+/+^/GT5^+/+^ floxP mice with C57Bl/6J background (age = 14 weeks, weight = 27 ± 2 g, *n* = 53). Animals for behavioral experiments were separated in following four groups; saline *floxP* (*n* = 8), *floxP* + HLS (*n* = 6), *Acan*KO (*n* = 6), *Acan*KO + HLS (*n* = 10), from which, additionally, IHC and WB analysis (both *n* = 4/group) was performed. Animals for acute IHC (*n* = 3/group/ time point) were used to observe the dynamic changes in synaptosome.

Global *Acan*KO mice (C57Bl/6J background) used for the experiment were bred by crossing the conditional knockout animals with *nestin-cre* (C57Bl/6J background) animals. For the experiment global *Acan*KO together with their littermate *floxP* mice were used (age = 14 weeks, weight = 27 ± 2 g, *n* = 32). Animals for behavioral experiments were separated in following four groups; *floxP* Global (*n* = 8), *floxP* global + HLS (*n* = 8), *Acan*KO Global (*n* = 6), *Acan*KO global + HLS (*n* = 7).

During behavioral study, mice were not marked to which treatment group they belong.

All experiments were performed in accordance with the European Communities Council Directive of 22 September 2010 (2010/63/EU), regarding the use of animals in research and were approved by the Ethics Committee of the Institute of Experimental Medicine ASCR, Prague, Czech Republic.

### ChABC injection

One week after MWM training period (Scheme 1) animals received ChABC (Sigma-Aldrich, Germany) or saline injections in to both hippocampi. Semiautomatic motorized operating system with mice 3D brain atlas (Neurostar, Tubingen, Germany), was used to drill the skull and inject the treatment within the pre-estimated coordinates to fully cover the volume of hippocampus. Animals were under isoflurane anesthesia, receiving local painkillers (mesocaine, subcutaneous 30 µl), shaved, and cut alongside sagittal suture to open 1 cm length window into the skin. System was accustomed on brain marks (Bregma, Lambda) and skull rotation, in order to predict injection path. With low *Z* axes speed (1 mm/min) the driller prepared 4 scull micro inserts for each hippocampus. With low speed the injector was set in the coordinates (AP, ML, DV- measured from bregma: L1 −1.58, −1.21, 1.59; L2 −2.54, −1.93, 1.74; L3 −2.7, −3.09, 3.1; L4 −3.52, −3.03, 3.52; P1 −1.58, 1.06, 1.67; P2 −2.54, 1.73, 1.65; P3 −2.7, 3.2, 3.21; P4 −3.52, 3.11, 3.47). Injection parameters were estimated; volume = 0.6 μl/injection, speed = 0.1 μl/min. After each injection the 3 min steady interval before taking out the injection needle was set, to prevent leaking. The skin was sutured and treated to prevent reopening (Novikov, Prague Czech Republic).

### Induction of local Acan knockout

Five weeks before MWM training *Acan* floxP animals received stereotaxic injections of AAV1-*hSynapsin-Cre* virus with the same stereotactic coordinates as in enzymatic study (*c* = 1 × 10^13^ u, volume = 0.6 μl/injection, speed = 0.1 μl/min/saline control) to knockdown production of aggrecan and destabilize PNNs in both hippocampi. The same operation parameters and stereotaxic coordinates were used as in case of ChABC treatment.

### AMP mediated hibernation like state

One week after ChABC injection (or 7 weeks after Acan knockout induction), the mice underwent HLS protocol (see in detail; [39]) Mice were intraperitoneally injected with 5’AMP (0.1 g/ml, 0.5 μl/g, Sigma-Aldrich) at room temperature. Body temperature was measured per rectum before injection and then in 15 min interval until the mice reached the room temperature (25 °C ± 1). The breathing depth and frequency were controlled. After reaching the room temperature the mice were placed in the stable cold environment (4 °C) and carefully watched. When the body temperature reached 18 °C, the time interval of 45 min necessary to induce synapse retraction was measured. Later the mice were placed at room temperature to passively warm up.

### FIB-SEM microscopy

#### Sample fixation and resin embedding

For FIB-SEM microscopy the mice were perfused with sodium cacodylate buffer (pH 7.4) followed by perfusing solution (2.5% glutaraldehyde/2% paraformaldehyde in sodium cacodylate buffer). The whole mouse brain was fixed for at least 5 h in 2% paraformaldehyde and 2.5% glutaraldehyde in 0.1 M CDS (cacodylate buffer) at a pH of 7.4. The fixed tissue was cut with vibratome (Leica VT1200) into 150 µm thickness slices and they were placed in 10 ml glass vials with fresh solution of fixatives. Brain slices were fixed for 1 h on ice. The samples were rinsed in 0.1 M CDS three times, 5 min each. Then the samples were stained with 1.5% (w/v) potassium ferrocyanide and 1% (w/v) osmium tetroxide in 0.1 M CDS for 30 min and next with 1% (w/v) osmium tetroxide in 0.1 M CDS for 30 min. After post-fixation and staining, samples were rinsed in distilled water three times, 3 min each. Then the samples were stained with 1% (w/v) uranyl acetate in distilled water for 30 min and rinsed in distilled water two times, 5 min each. Brain slices were dehydrated in graded ethanol (EtOH) series, 2 min each change (1 × 30%, 1 × 50%, 1 × 70%, 1 × 95%, 2 × 100%), in 100% EtOH 5 min. The dehydration was followed by embedding in mixture of Epon EmBed 812 hard with EtOH (1:1) for 30 min and then in 90% Epon EmBed 812 hard an overnight on rotator. Then we changed Epon with the fresh one (100%) and agitated slowly for 4 h. Finally, the sections were placed on glass microscopes slides coated with mold separating agent using wooden cocktail sticks and covered with fresh 100% Epon and placed in 60 °C oven for 24 h.

#### Preparing the sample for the FIB-SEM

After polymerization, the resin layer containing the samples was separated from between the two glass microscope slides and washed thoroughly to remove any mold separating agent. Using a transmitted light microscope (Carl Zeiss Axiozoom.V16) we identified the region of interest on mouse brain slices within the slice of resin. A small (5 mm × 5 mm) square around the region of interest was cut using a razor blade and stuck to the top of blank resin block with acrylic glue. After the glue hardening the block was mounted into holder of the ultramicrotome (Leica EM UC7) and trimmed a small pyramid around the region of interest with a razor blade. Then we trimmed the block surface with glass knife until the embedded tissue will appeared on the resin surface. Finally, the trimmed block was cut away from the remaining resin stub to ensure that only a small block is placed inside the FIB-SEM. The small epon block was mounted on a regular SEM stubs using conductive carbon and coated with 25 nm of platinum (using High Vacuum Coater, Leica ACE600).

#### FIB-SEM imaging

Ion milling and image acquisition was performed in Dual beam system FEI Helios NanoLab 660 G3 UC.

By using a low magnification and secondary electron imaging (20 kV and 0.8 nA) the block was oriented into best position and the region of interest was chosen. The protective layer (~1 µm thick) of platinum was deposited, using the gas injection system of the microscope, onto the surface of the block, above the region of interest. A large trench around protective region was milled at a current of 21 nA and 30 kV by focus ion beam. It was followed by fine milling at 0.79 nA and 30 kV, thickness of slices was 90 nm. The SEM imaging of the milled face (area of interest) was done in backscattered imaging mode and the serial SEM images were acquired at 2 kV and 0.2 nA using an InColumn backscattered electron detector (ICD). The region of interest was the dendritic layer below hippocampal CA1 area, determined based on preview of the low magnification images. 66 images of 9216 by 6144 nm (image 3072 × 2048 pixels, the XY pixel size was set to 3 nm) were analyzed per time point (37 °C, 16 °C–0 h, 37 °C–24 h) at each group (saline, ChABC).

Synapses were counted using FIJI (ImageJ) when: a pre- and postsynaptic density was present and vesicles in the presynaptic area detected.

### Immunohistochemistry

#### Tissue isolation and preparation

For immunohistochemistry, the mice were transcardially perfused with PBS followed by paraformaldehyde (4% PFA in PBS). The brains were left in PFA overnight then gently washout in PBS, 20% sucrose and then 30% sucrose (each solution time = 24 h, *t* = 4 °C). Using cryotome 20 µm thick coronal sections were prepared.

#### IHC staining and visualization

To evaluate the effect of treatments on PNNs and synaptic input of parvalbumin positive inhibitory interneurons, immunohistochemical staining on series of 20 μm coronal brain section was performed.

To observe integrity of PNNs and monitor the effect of ChABC or AAV1*hSynapsinCre* local knockout staining biotinylated *Wisteria floribunda* agglutinin (WFA, 1:150, Sigma, Germany), and anti-aggrecan primary antibody (1:150, Sigma) together with anti- parvalbumin primary antibody (1:500, Synaptic Systems, Gottingen, Germany) were used. For analysis of parvalbumin positive inhibitory interneurons synaptic connectivity, antibodies against parvalbumin (1:500, Synaptic Systems), PSD95 (excitatory postsynaptic, Synaptic Systems), gephyrin (1:250, inhibitory postsynaptic, Synaptic Systems), bassoon (1:250, excitatory presynaptic active zone, Synaptic Systems), vGLUT1 (1:200, excitatory resynaptic, Synaptic Systems), and vGAT (1:200, inhibitor presynaptic, Synaptic Systems) were used. To visualize primary antibodies positivity anti-mouse Alexa Fluor 594 (1:200, on PV neurons) and anti-rabbit Alexa Fluor 488 (1:300, on gephyrin, bassoon, vGAT) secondary antibodies were used. To visualize the primary antibody a streptavidin 488. Alexa Fluor 488 and Alexa Fluor 594 secondary antibodies were used (goat anti mouse 1:300, goat anti rabbit 1:200, streptavidin AF488 1:300).

#### Confocal imaging and super resolution microscopy

To evaluate synaptic markers, present on parvalbumin^+^ inhibitory interneurons in CA1 region of hippocampus, confocal microscope Zeiss880 Airyscan, in supper resolution mode, was used. Pre-synaptic markers- only (Fig. S3), were measured on the ×63 oil immersion objective (Plan-Apochromat ×63/1.4 Oil DIC M27, zoom 1.5, pixel dwell 0.82 μs, *x*: 1272, *y*: 1272, *z*: 58, channels: 3, 12-bit). To quantify synapses on PV neurons (Figs. 1 and 2), co-location measurement on the ×100 oil immersion objective (alpha Plan-Apochromat ×100/1.46 Oil DIC M27 Elyra) was used with neuron area selection based on zoom function (3 −5 x zoom, depending on the neuron size). Airyscan mode with 32 detectors signal magnification and highest super-resolution setting (SR mode) and 3 separate channel measurements were used. For super resolution mode, laser power was maintained below 13%. To quantify the synapse numbers, colocation of pre- (vGlut1/vGAT, 594 nm), postsynaptic (PSD95/Gephyrin,488 nm) and PV^+^ signal (405 nm) was evaluated. To visualize intensity and structure of PNN signal by WFA and aggrecan antibody staining, ×10 objective (plan-achromat ×10/0.45 M27, zoom 0.7, Pixel dwell 0.77 μs, 1214 × 1214 × 22, Average- line 2, 12-bit) and ×60 oil immersion objective (Plan-Apochromat 63/1.4oil DIC M27, zoom 1.5, Pixel dwell 0.82, 1272 × 1272 × 56, Average- line 2, 12-bit) were used. Lasers- track 1 (488 nm, first detector), track 2 (561 nm) and track 3 (405 nm) (both second detector) with two detectors setting. Laser power was maintained below 3%.

For the quantification of the synaptic markers, present on parvalbumin^+^ inhibitory interneurons these basic parameters were used; *n* = 3–4 animals/group, 10 neurons/animal (Fig. S3) (CA1 region) (confirmation of co-location 2–3 neurons per animal Fig. 2B–I), at least 20 sections/neuron).

### Western blot

#### Tissue isolation and preparation

For western blot analysis the tissue was prepared on ice within the tissue lysate solution (ddH_2_O, Tris 50 mM, NaCl 150 mM, EDTA 2.5 mM, Triton X 1%, sodium deoxycholate 0.1%, PhosSTOP and Complete- mini EDTA-free (Roche, Germany)). The brain was cut to hemispheres and the hippocampus isolated and placed in to the 200 µl Eppendorf tubes with 100 μl of TLS. Tissue was mechanically homogenized, vortexed and centrifuged at 4 °C and 5000 × *g* for 30 min. After isolation, the supernatant was collected, and the protein concentration measured using BCA assay. The tissue lysates were placed at −80 °C until use.

#### Electrophoresis, western blot and immunoprecipitation

To confirm the synaptic changes after enzymatic or genetic manipulation of PNNs and hibernation like state. Series of pre and postsynaptic markers were measured. To separate proteins the Tris-glycine 4–15% precast gel on BioRad miniprotean aperture was used. Transfer was done on PVD membrane. Applied antibodies against postsynaptic markers: PSD95 (1:1000, Abcam, Cambridge, UK), GAD65/67 (1:3000, Abcam). Applied antibodies against presynaptic markers: vGLUT1 (1:1000, SYSY), vGAT (1:1000, Synaptic Systems), SNAP25 (1:1000, Synaptic Systems). Secondary antibodies goat anti rabbit-HRP (1:15,000, Abcam), rabbit anti mouse –HRP (1:15,000, Abcam) and goat anti-guinea pig-HRP (1:5000, Abcam) were used to visualize proteins of interest.

### Behavioral tests

#### Morris water maze

Morris water maze test for long term memory was applied to determine the impact of ChABC or local Acan removal in the hippocampus in model of AMP mediated hibernation like state synapse retraction. Interval of 60 s was given for animal to reach the target platform (at least 0.5 s interval at the target zone). When not reaching it, the value was automatically estimated as 60 s. Training period was for 5 consecutive days, four trials per day, each from different side of the pool (West, North, East and South). The order of release sites was changed every day to prevent scheme orientation. Two visible permanent cues were present. First day of training period mice were placed in to the MWM swimming pool with visible target platform. From the second day of training period the platform was hidden under the level of water (~0.5 cm). Two weeks after training period, including stereotaxic injections of enzymatic treatment and hibernation like state protocol, the mice started experimental MWM period. Again, five consecutive days of four trials per day from four distinct locations.
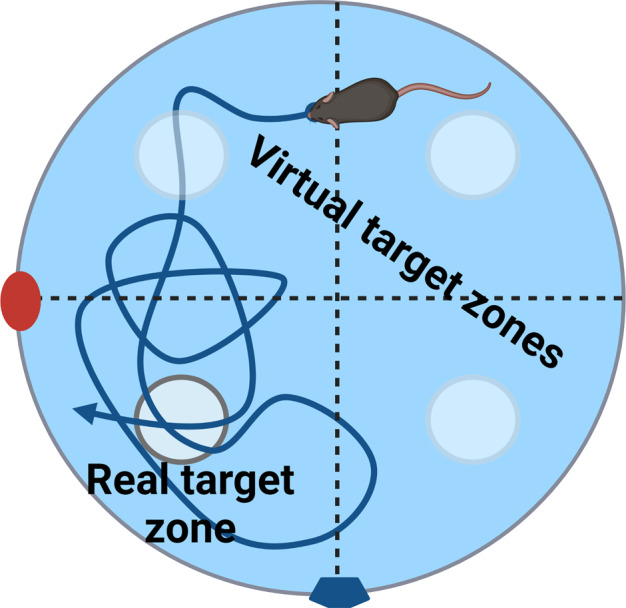


#### Probe test

Probe test trials were applied to define the current state of memory of MWM task, and by comparison with the previous time points the ability to re-/learn the target position. The target platform was removed, and animals were placed from east entrance in to the pool for 60 s trajectory analysis. Four probe test trials held on day 1 (naïve animals), 6 (trained animals), 22 (memory retention test), 27 (re-learning ability.) were used to determine animal progress.

#### Spontaneous alternation/Y-maze

Spontaneous alternation test in Y-maze was used at the end of experimental period to confirm no pathological changes in explorative behavior in new, experiment non-related arena. Mice were placed in the middle of triangular platform and the trajectory and entrance in the three distinct target zones were measured:

$${\rm{Spontaneous}}\,{\rm{alternation}}\% = \left( {\# {\rm{spontaneous}}\,{\rm{alternation}}{{{\mathrm{/}}}}\left( {{\rm{total}}\,{\rm{number}}\,{\rm{of}}\,{\rm{entries}} - 2} \right)} \right) \times 100$$

### Statistic

Using Sigma-stat software the study data were evaluated. For statistical evaluation of behavioral data, where the same subjects have been continuously assessed, the two-way repeated measurement ANOVA test was used (probe tests in enzymatic group TW-ANOVA (missing values day 22)). For confocal microscopy measurements, FIB-SEM microscopy and proteomic analysis (Western blot, WES), where treatment and time factor were considered, the two-way ANOVA was applied. Tukey post hoc multiple comparison test was used to study detail interactions, Holms-Sidak test was used to compere experimental data to control. Data presented in the graphs are expressed as arithmetic mean, with standard error of mean intervals. The normality of the tested values and statistical significance of the differences among groups in the text are described by *F* (H, q) values and *p* values, respectively. In the text and graphs when *p < 0.05, **p < 0.01, ***p < 0.001. Statistical color coding: between different time points the asterisk color coding indicates in which group the statistical significance was found. Within the single time points, the asterisk color coding indicates to which group the statistically significant difference was detected.

## Supplementary information

Supplements
