# Supplementary material for: Perineuronal nets affect memory and learning after synapse withdrawal
Source: Transl Psychiatry. 2022 Nov 15;12:480. doi: 10.1038/s41398-022-02226-z (PMC9666654; doi:10.1038/s41398-022-02226-z)
Supplement: Supplementary file 1 — Supplements [file 41398_2022_2226_MOESM1_ESM.pdf]

Supplements

Injection site

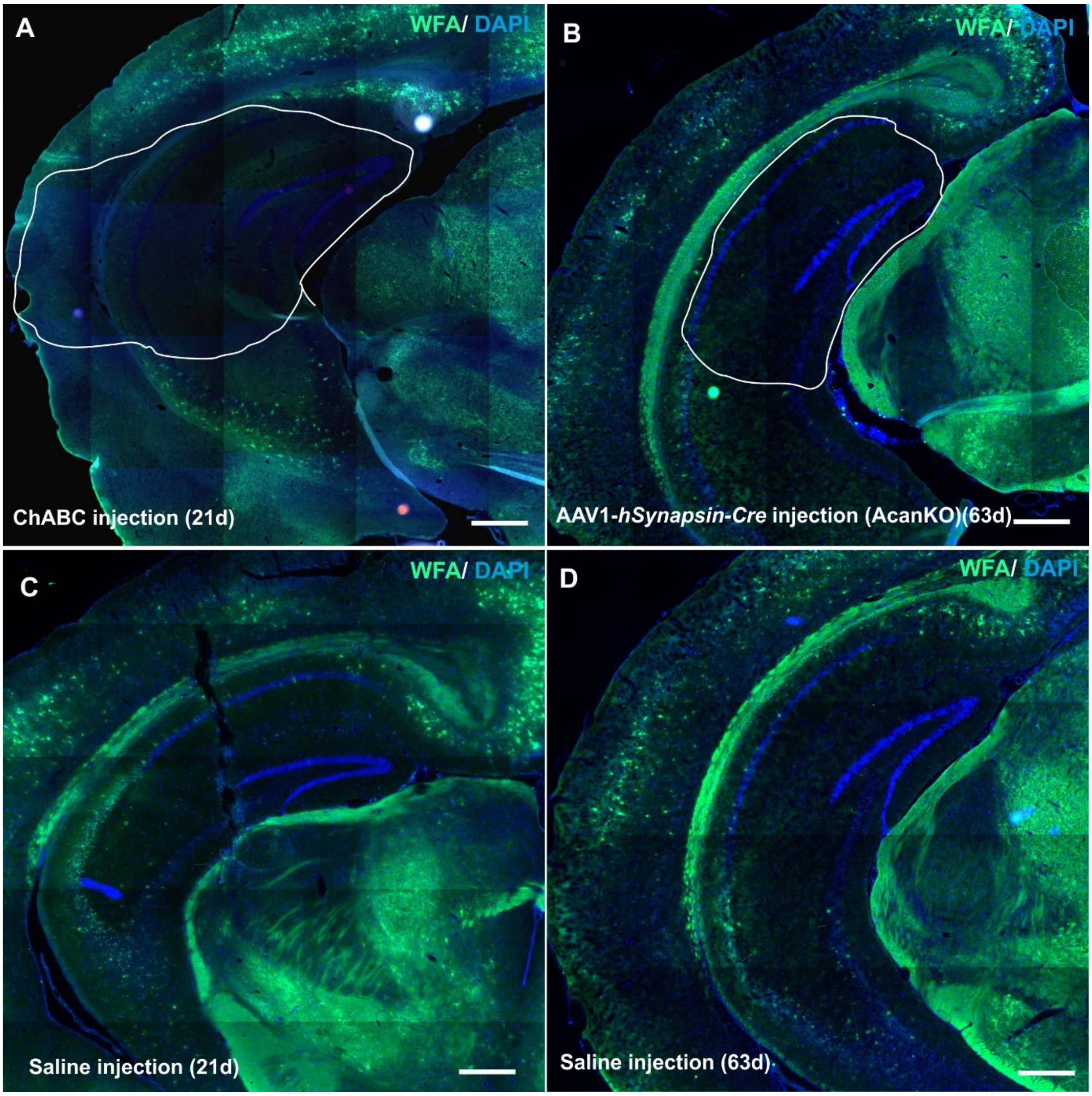

Figure S1. Injection site of ChABC (A,21 days after injection) and AAV1-*hSynapsin-Cre* AcanKO (B, 63 days after injection) at the end of the study (Day 28, Scheme1). Control injection sites showing no PNN digestion (WT- Saline (C), floxP - saline (D)). Stained on WFA (green) and DAPI (blue). Scale bar 500um.

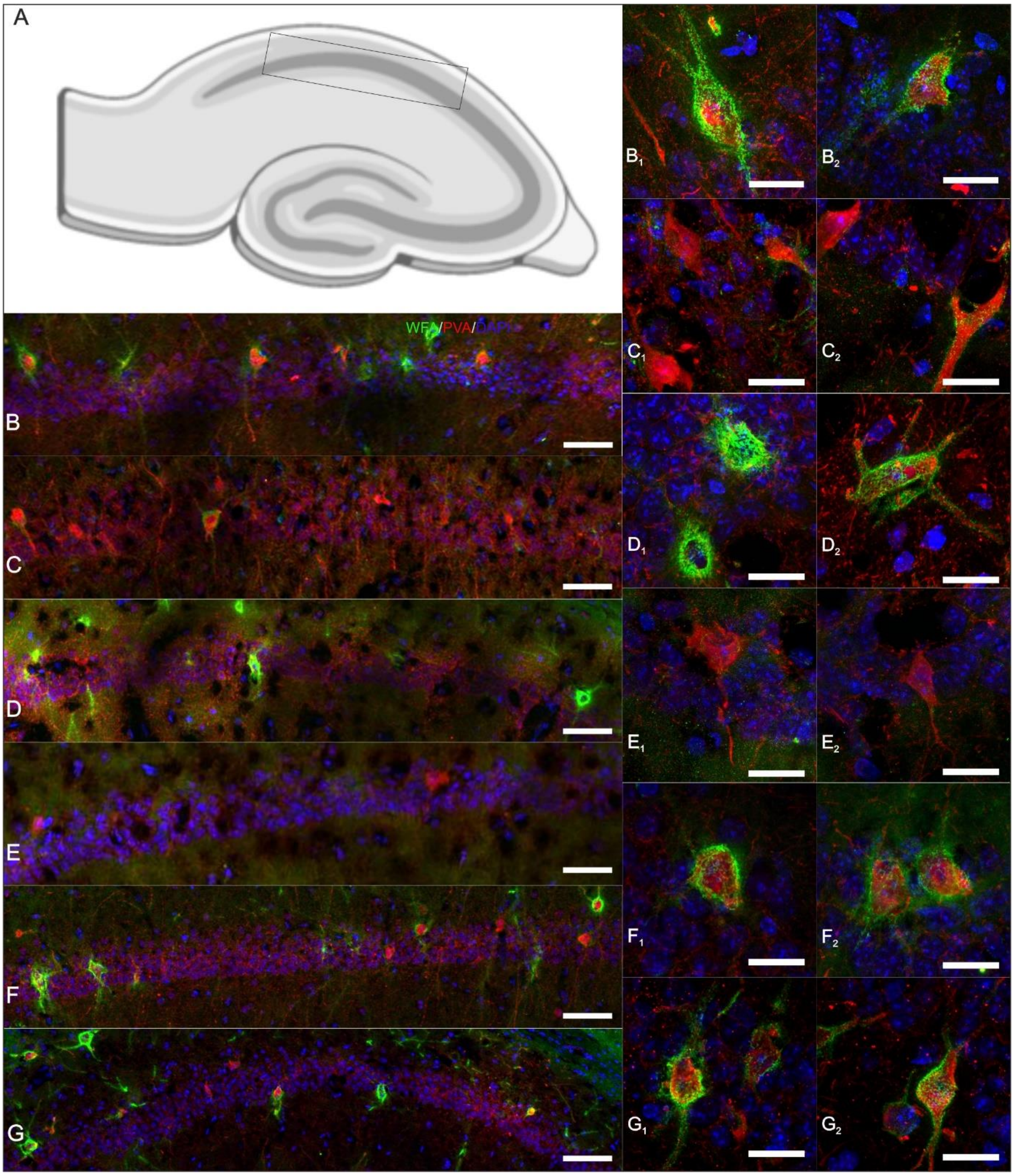

Figure S2. Detail image of PNN disintegration in the CA1 are of hippocampus (Illustrated location, A) of enzymatically treated (ChABC C, detail C1,2) or AcanKO animals (E, detail E1,2) at day 8, before HLS. In CA1 area of saline treated animals no sign of disintegration was observed (Saline group B, detail B1,2, floxP group, D detail D1,2). Intact WFA positive PNNS were observed in saline treated WT (F, Detail F1, 2) or floxP (G, detail G1, 2) mice that underwent HLS procedure. Samples were taken immediately after synapse withdrawal incubation period when BT was 16°C. WFA (Green) Parvalbumin (Red) DAPI (Blue). Scale bar 100μm (A - F), 10μm (A 1 ,2-F 1, 2).

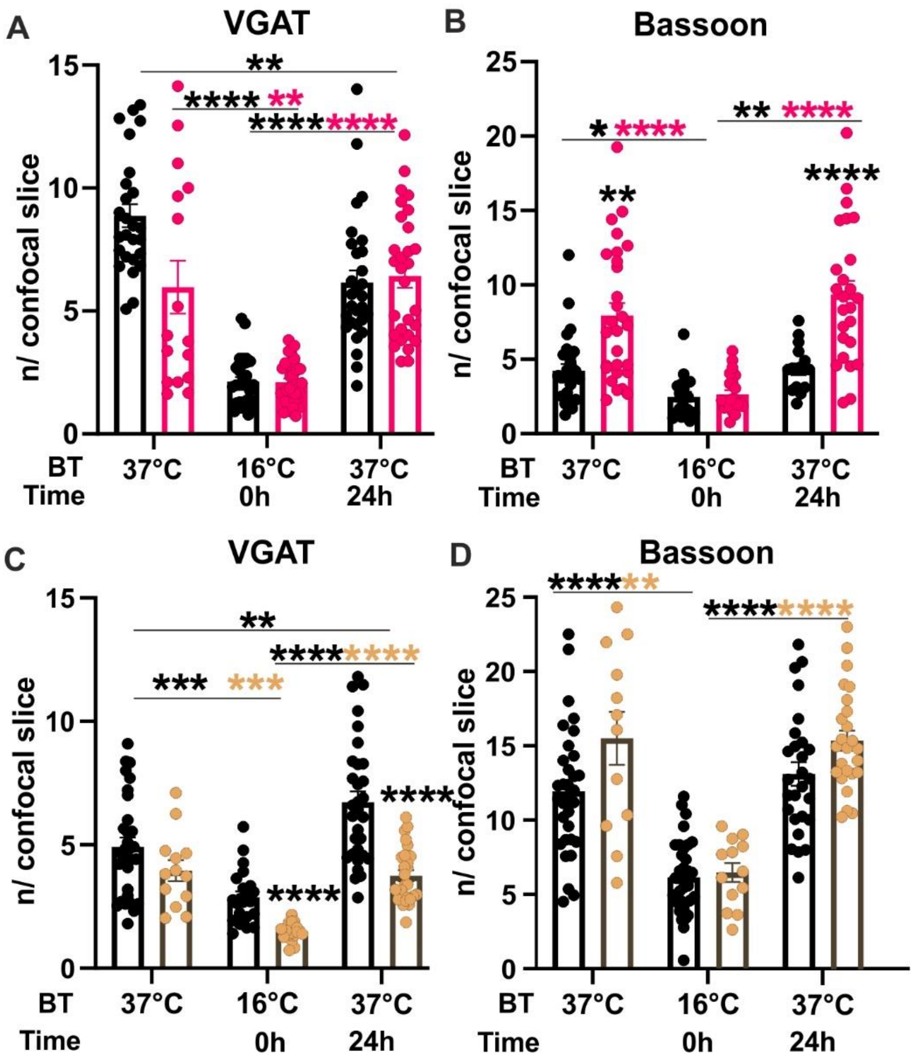

Figure S3. Number of excitatory and inhibitory inputs ((presynaptic terminals VGAT/ BASSOON) all on CA1 PV+ neurons) was measured before HLS. Immediately after HLS and 24h after HLS. Individual presynaptic components localised on PV+ signal was evaluated on single neuron confocal image Z-stack series and newly appearing markers per slice were counted. Similarly, to collocational synaptic analysis (Fig2) both inhibitory and excitatory presynaptic terminals have decreased immediately after HLS and then recovered within 24h, (B) In case of ChABC, Inn coherence with figure2, prior HLS and 24h after HLS, significant increase in Bassoon terminals was observed. (C) In *Acan*KO, immediately and 24h after HLS significant decrease in VGAT terminals was observed. * p<0.05, **p<0.01, ***p<0.001 (for statistic, see stat table S3,4 in attachments). (Displayed as individual neuron averages per slice, n= 10-12 neurons per animal, 3-4 animals/time point/group).

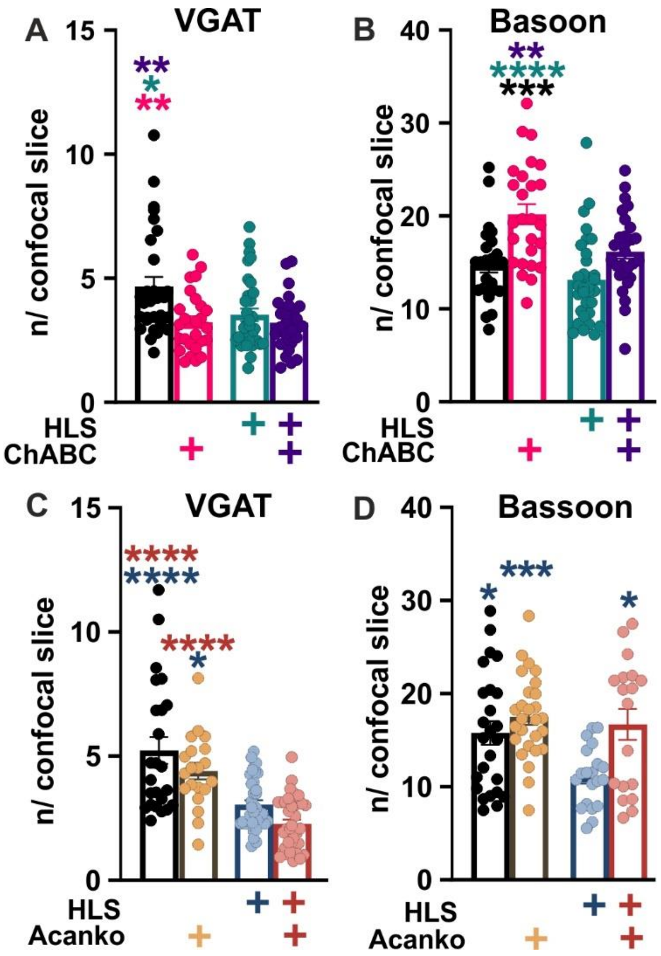

Figure S4. Number of excitatory and inhibitory inputs (all on CA1 PV⁺ neurons) was measured on day 28. Individual presynaptic components localised on PV+ signal was evaluated on single neuron confocal image Z-stack series and newly appearing markers per slice were counted. All treatments, HLS, ChABC and their combination showed decreased amount of vGAT⁺ pre-synaptic elements in comparison with saline treated animals (**A**). Animals with digested PNNs have shown significantly higher number of bassoon⁺ pre-synaptic elements, when compared to other treatment groups (B). Moreover, the HLS animals pre-treated with ChABC has shown significantly prevented reduction in comparison with HLS only (**B**)In the transgenic animals, strong effect of HLS was observed. The HLS condition led to and significant reduction of vGAT positive presynaptic elements, when compared to non-cooled control groups. Both AcanKO groups had lower amount of vGAT⁺ elements than related floxP groups and cooling further reduced these numbers (**D**). Additionally, the HLS treated floxP animals had significantly lower number of bassoon (B) than all the other groups, even the AcanKO + HLS. Statistical significance was marked * p<0.05, **p<0.01, ***p<0.001 (for statistic, see stat table S3,4 in attachments). (Displayed as individual neuron averages per slice, n= 10-12 neurons per animal, 3 animals/time point/group).

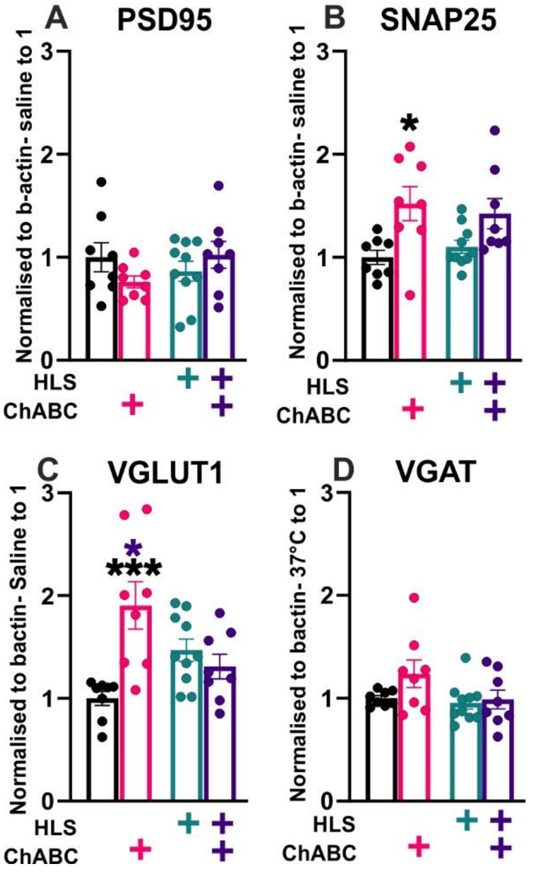

Figure S5. Western blot analysis of synaptic proteins in all experimental groups at the end of experiment has revealed trend in lover levels of postsynaptic proteins (PSD95) after HLS partially compensated by ChABC pre-treatment. In presynaptic markers (vGLUT, vGAT and SNAP25) significantly higher protein levels were observed in ChABC treated animals; expression of SNAP25 marker was increased even after HLS state. Statistical significance was marked * $p<0.05$, ***$p<0.001$ (for statistic, see stat table S5 in attachments). Illustrative blot images in supplement. (Displayed as individual brain sample values , n= 6-8 samples per group).

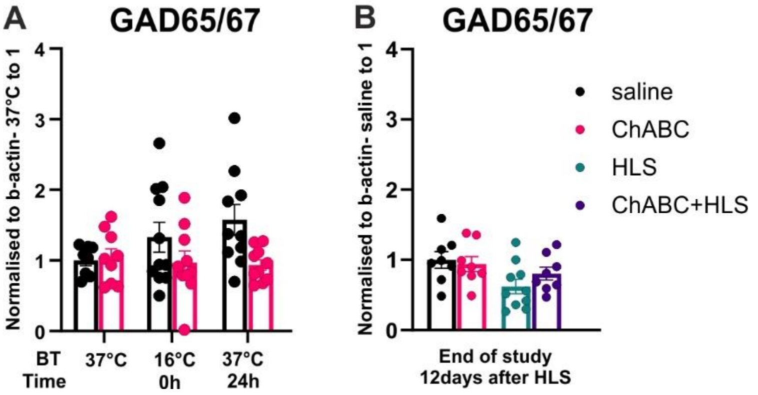

Figure S6. Western blot analysis of levels of the GABA synthesis enzyme GA65/65 before, immediately after and 24hrs after HLS(A) and at the end of behavioural study (B) (for statistic, see stat table S6 in attachments). Illustrative blot images in supplement. Displayed as individual brain sample values , n= 7-10 samples per group).

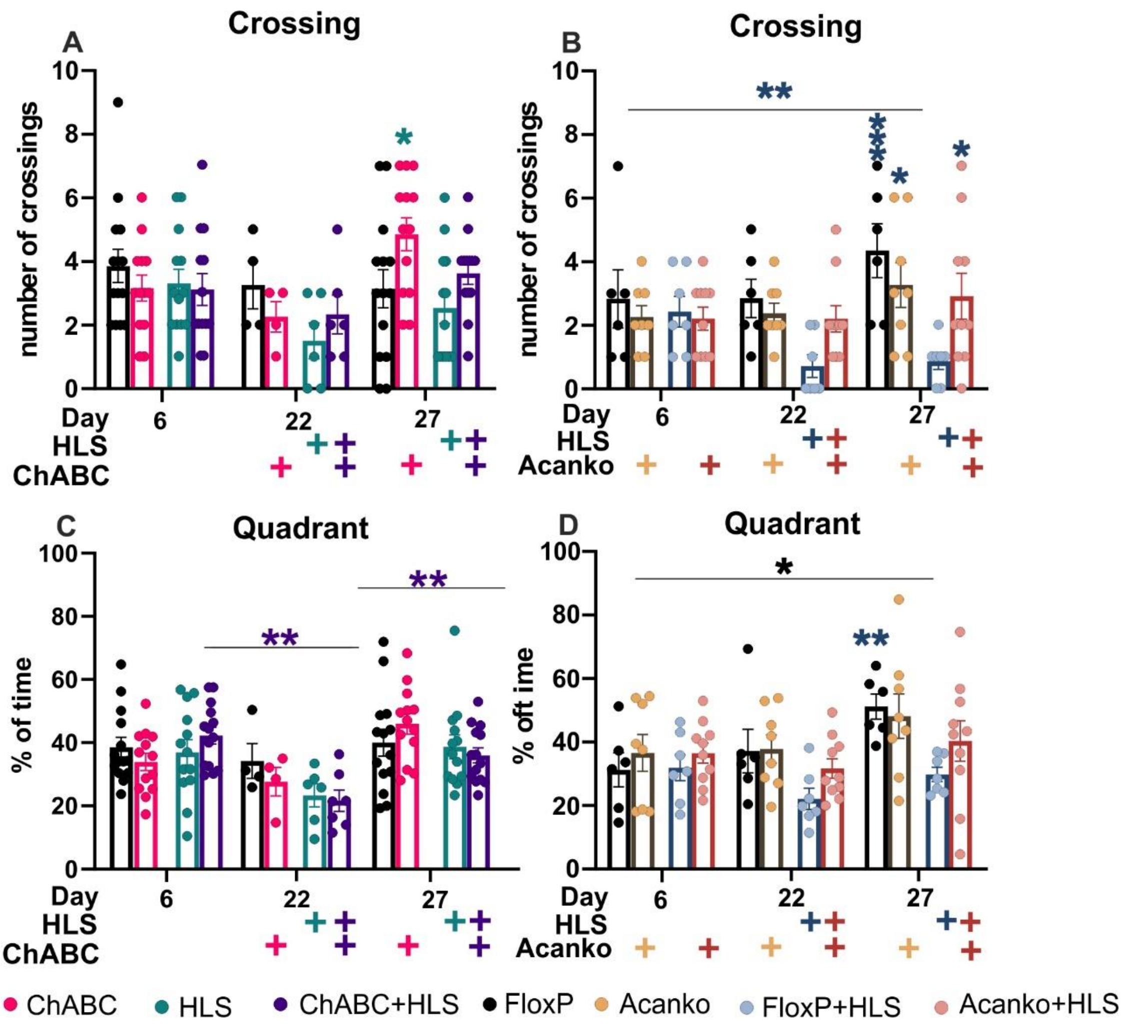

Figure S7 Probe test of Morris water maze showing decreased crossing of target zone (A, B) (**B** Crossing TW-RM-ANOVA Group F (3,27) = 5.77 p=0.0035, FloxP vs. FloxP HLS q=5.735 p=0.002, AcanKO vs FLoxP HLS q=3.981 p=0.042) and reduction of target quadrant preference (C, D) (D, Quadrant TW-RM-ANOVA Group F (3,27) =3.353 p=0.0335, AcanKO vs FloxP HLS q=4.133 p=0.0331)after HLS. FloxP animals have shown no recovery in target crossing after HLS. Whereas, AcanKO animals have shown no visible deficit in target crossing in re-learning week. Statistical significance was marked * p<0.05, **p<0.01, ***p<0.001 (for statistic, see stat table S7 in attachments). (Saline n=13, HLS n=14, ChABC n=13, ChABC HLS n=14, FloxP n=8, FloxP HLS n=6, *Acan*KO n=6, *Acan*KO HLS n=10)

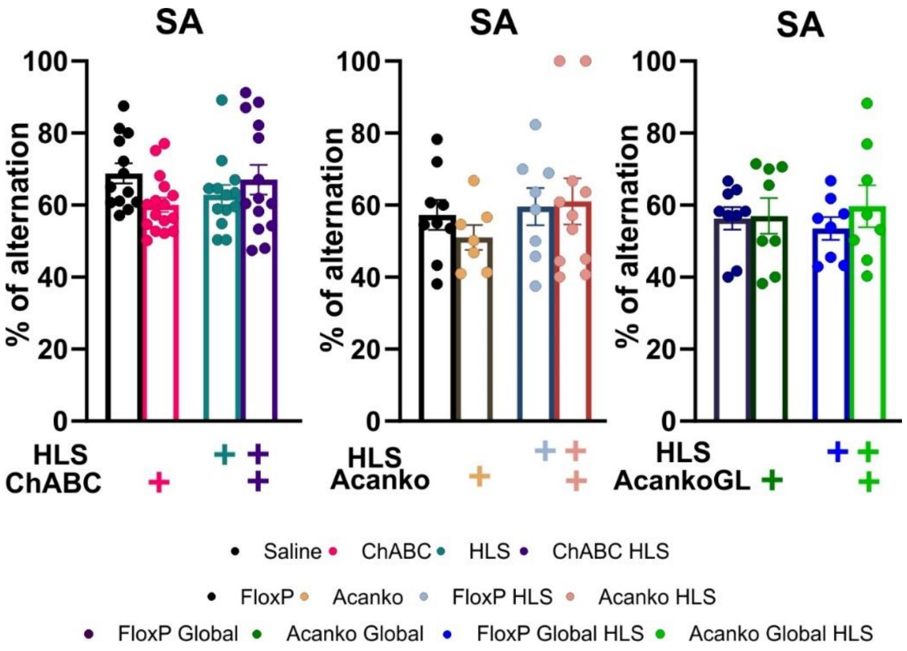

Figure S8. Spontaneous alternation test performed at the end of the experiment (day 28). There were no significant differences between groups in working memory or general activity in the maze between treatment groups. All animals were showing values within the range of healthy individual.

((Saline n=13, HLS n=14, ChABC n=13, ChABC HLS n=14; FloxP n=8, FloxP HLS n=6, *Acan*KO n=6, *Acan*KO HLS n=10 ; *floxP* Global (n=8), *floxP* global + HLS (n=8), *Acan*KO Global (n=6), *Acan*KO global+ HLS (n=7))

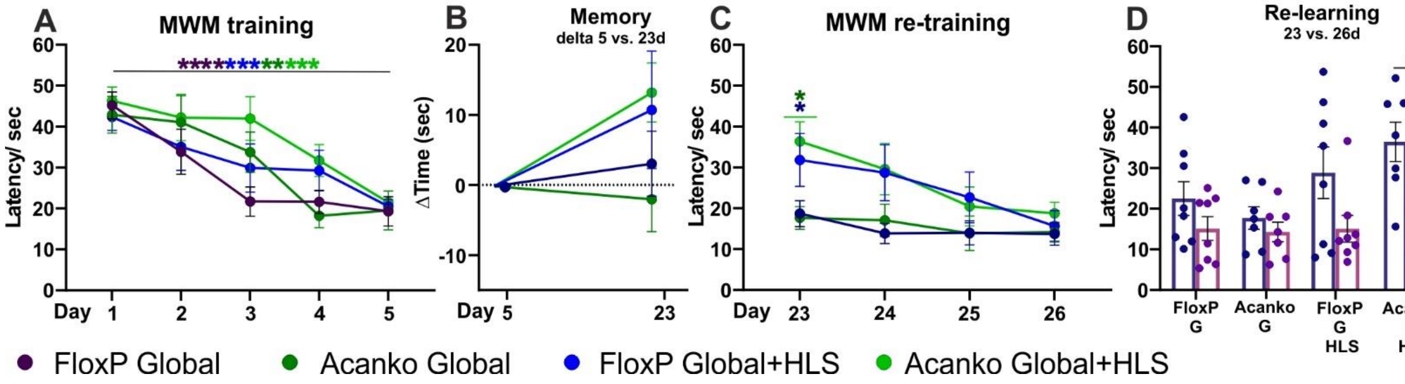

Figure S9. Morris Water maze; effects of whole brain aggrecan knockout on memory before (A) and after HLS (B-D). Global Aggrecan knockout was prepared by crossbreeding of FloxP mice with Cre-Breeder. All, FloxP and AcanKO Global mice showed normal learning (A). The training week was followed by HLS, then memory testing and training resumed on day 23. Hibernated animals showed a partial loss of memory, but not to the level of naïve animals (B). This loss was not altered in AcanKO Global $^{+}$ HLS mice (B). During the relearning phase (C, detail analysis D), animals in the FloxP Global HLS group did show strong trend in re-learning. This became statistically significant in animals treated with Acanko Global and HLS (D). Significance * p<0.05, **p<0.01, ***p<0.001. (for statistic, see stat table S9 in attachments) . (*floxP* Global (n=8), *floxP* global + HLS (n=8), *Acan*KO Global (n=6), *Acan*KO global+ HLS (n=7))

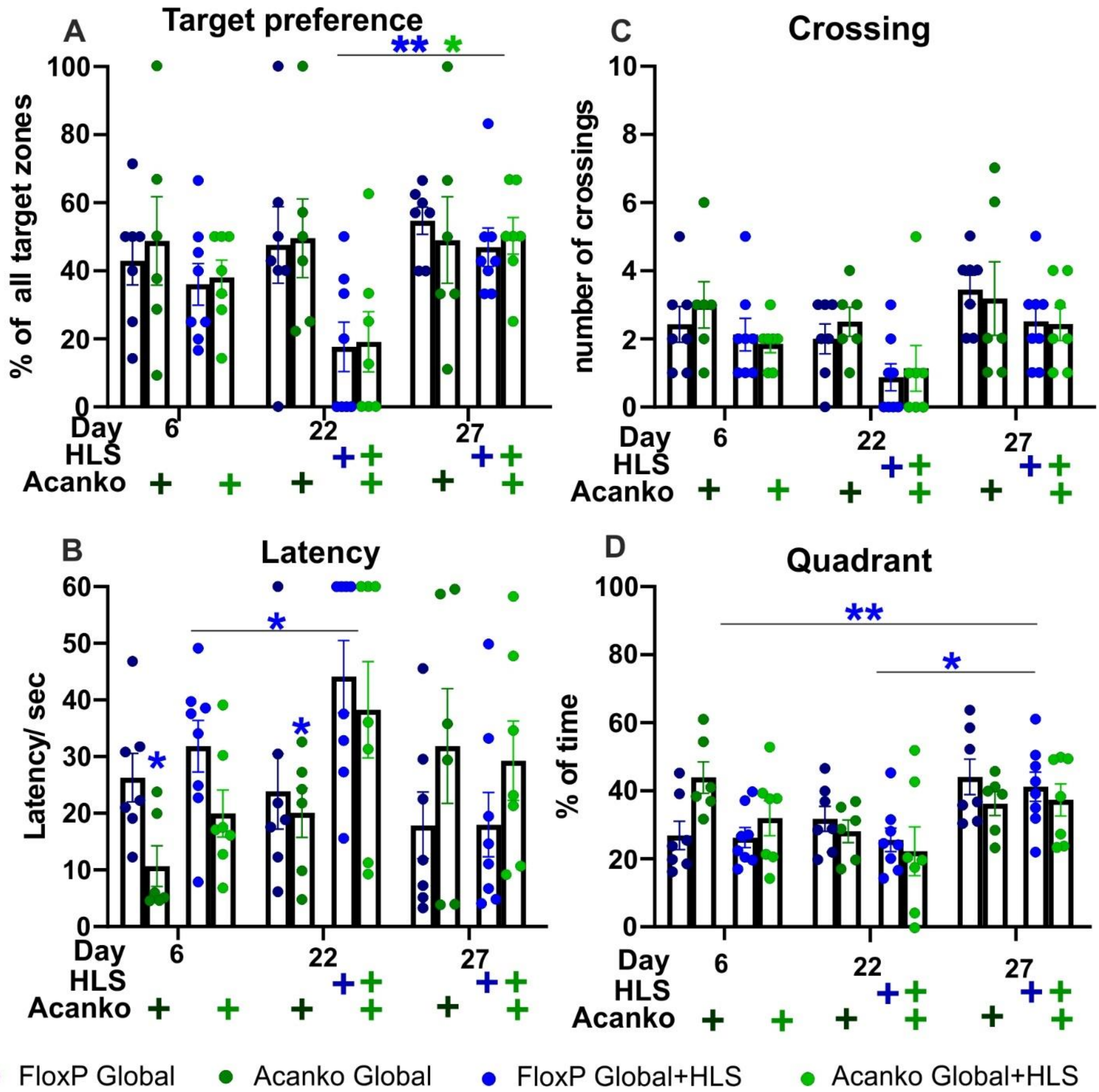

Figure S10. Within the probe tests to MWM a significant impact of HLS was observed at day 22, within the re-learning week almost a full recovery was observed. No impact of the Acanko Global modifying the HLS was observed on the day 22 and 27. In detail, significant increase in latency to reach the target position was found after HLS (B). Significance * p<0.05, **p<0.01, ***p<0.001. (for statistic, see stat table S10 in attachments. (*floxP* Global (n=8), *floxP* global + HLS (n=8), *Acan*KO Global (n=6), *Acan*KO global+ HLS (n=7))

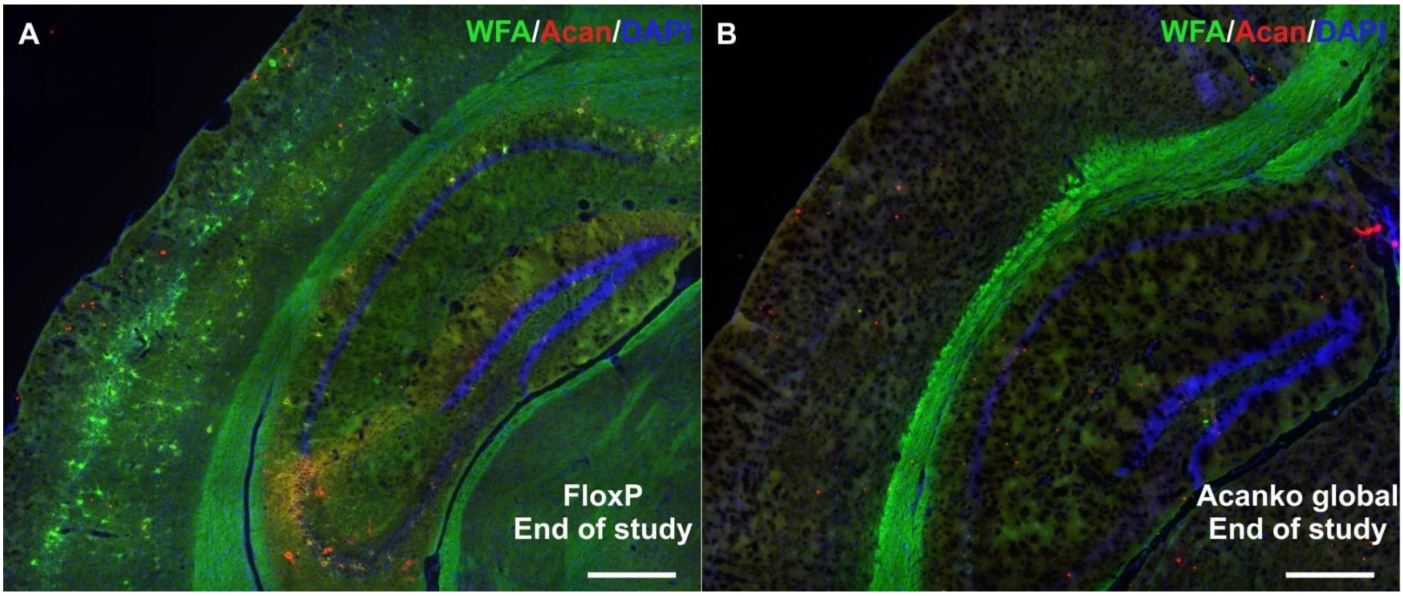

Figure S11. Example of FloxP (A) and Acan Global knockout (B) brains at the end of the study (day 28), showing complete reduction of WFA and Aggrecan based PNNs after crossbreeding FLoxp Mice (GT5 $^{+/+}$/GT3$^{+/+}$) with Nestin Cre Breeder ($^{+/+}$). Acanko Global mice are lacking the nets not only in hippocampal structures, where was focused main part of the project, but also in cortical regions, including medio lateral cortex. WFA (green) Aggrecan (Acan, red) and DAPI (blue) are visualised within the figure. Scale bar 500μm.

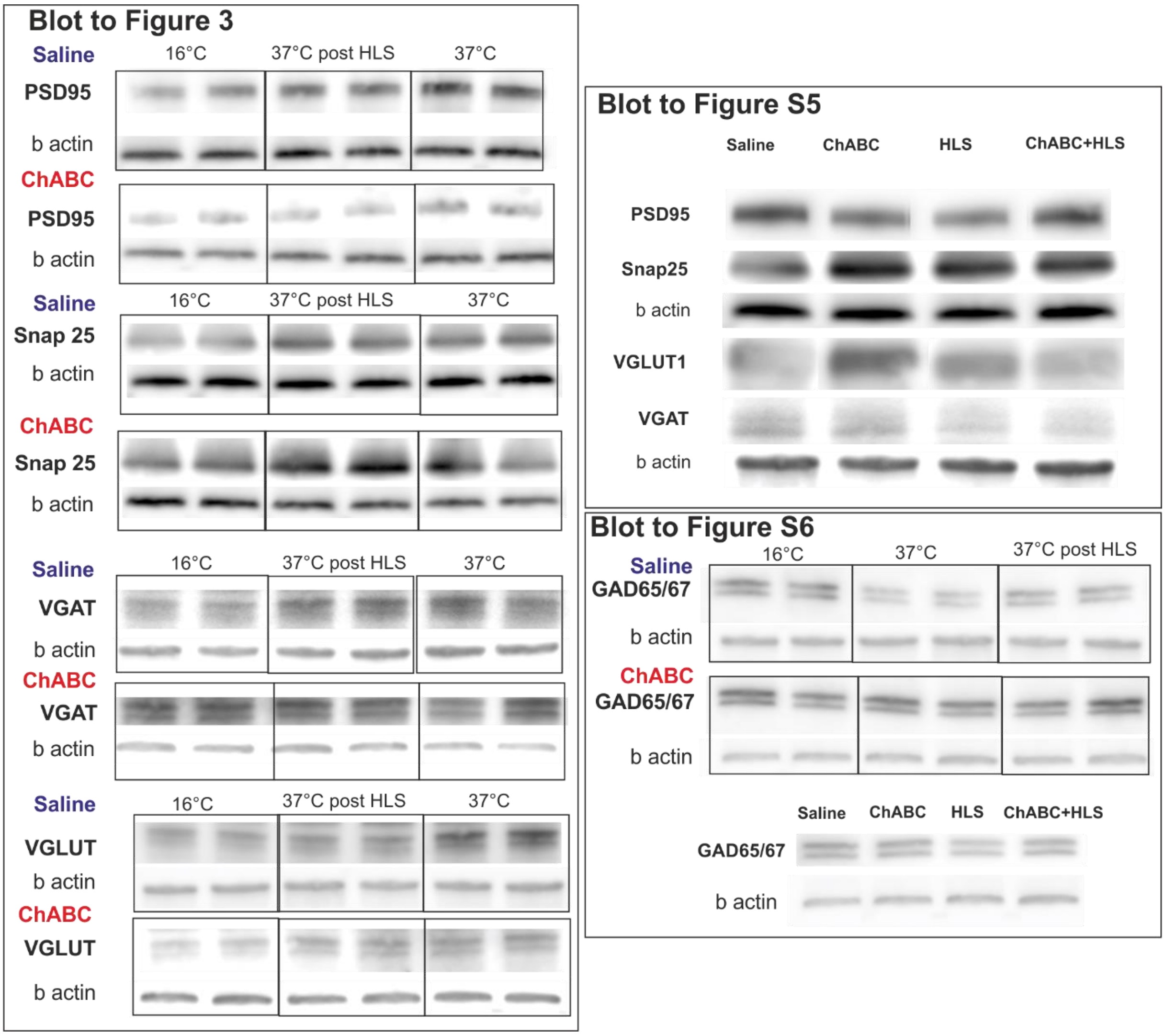

Figure S12. Western blot examples to Figure 3, S5 and S6. Example of protein levels of tested markers before, immediately and 24h after HLS in Saline and CHABC group (Figure 3). Example of protein levels of tested markers at the end of the behavioural study in Saline and CHABC group (Figure S5). Example of protein levels of GAD65/67 markers before, immediately and 24h after HLS and at the end of behavioural study, in Saline and CHABC group (Figure S6).

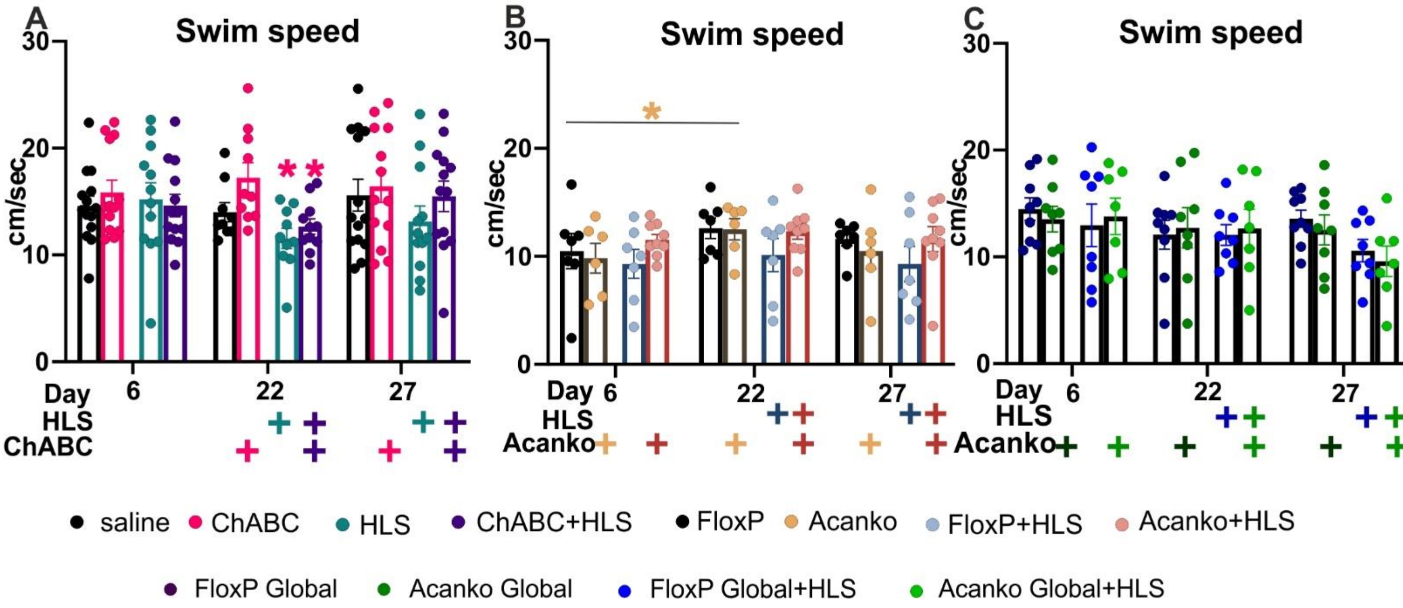

Figure S13 Probe swim velocity. In all groups the swim speed was measured at 6th ,22nd and 27thday probe tests. No statistically significant decrease over time was noticed in any of the tested groups; (A) enzymatic (Two-Way ANOVA F (3, 48) =2.245 p= 0.0951), (B) local AcanKO (Two Way-RM ANOVA F (3, 25) = 1.314 p= 0.2918), or (C) Global AcanKO study (Two Way-RM ANOVA F (3, 28) = 0.5156 p= 0.6749). Local differences at day 22 were found in comparison to ChABC treated animals (A, ChABC vs. HLS q=4.798 p=0.0176, CHABC vs. ChABC HLS q=4.183 p =0.0466).However, no significant diference was found in comparison of any of experimental groups to saline injected control animals. In Local AcanKO study the KO mice shown increase in velocity from 6 to 22 day (B, ChABC 6 vs. 22d q=4.843 p=0.0417). Significance * p<0.05. ((Saline n=13, HLS n=14, ChABC n=13, ChABC HLS n=14; FloxP n=8, FloxP HLS n=6, *Acan*KO n=6, *Acan*KO HLS n=10 ; *floxP* Global (n=8), *floxP* global + HLS (n=8), *Acan*KO Global (n=6), *Acan*KO global+ HLS (n=7))

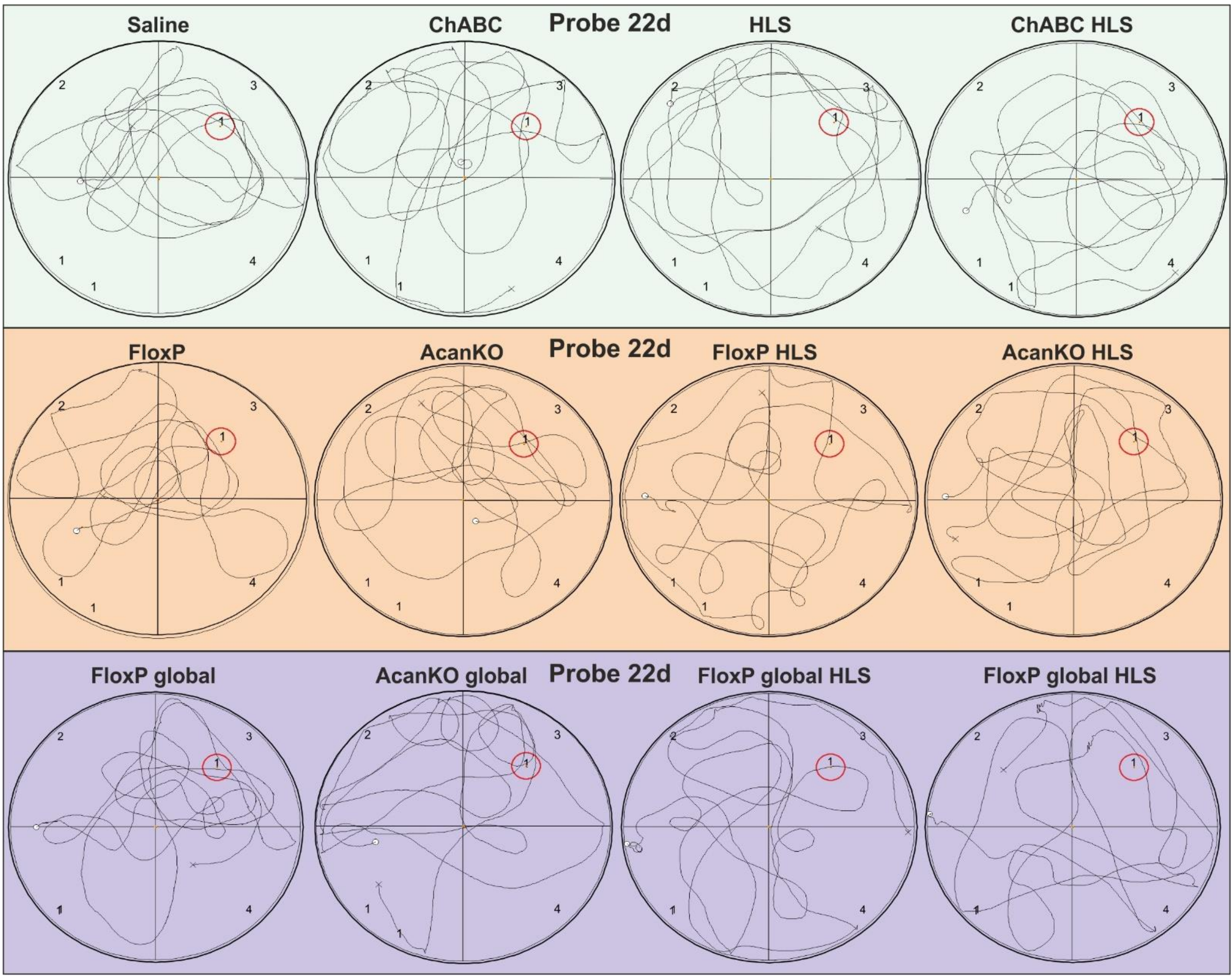

Figure S14. Examples of trajectory at 22nd day probe tests (animals corresponding to average value displayed in the graphs of Figure 5, S7, S10). Trajectories are showing active pattern of searching for the position of removed hidden platform.

Statistic to Figure 1	EM	37	16	37post
One way ANOVA	37		q=5.606	q=5.485
	16	0.0005		q=9.855
	37post	0.0007	0.0001	
	Vgat/Gephyrin	37	16	37post
	37		q=5.384	x
	16	0.0208		q=4.252
	37post	ns	0.0358	
	Vglut/ PSD95	37	16	37post
	37		q=7.137	x
	16	0.0037		q=5.848
	37post	ns	0.0106	
**Training**	Saline: 1 vs5	q=7.566 P<0.0001	HLS: 1 vs 5	q=7.709 P<0.0001
**experiment**	23	ns	23	x
Group	24	ns	24	x
F=4.701, P<0.05	25	ns	25	x
	26	0.0411	26	t=2.613
**Delta**	**Saline vs HLS**	**HLS**	5	23
group	ns	5		t=2945
F(1,24) =4.338, P=0.0481	t=3.109 P<0.01	23	P=0.0099	
**re-learning**	**Saline vs HLS**	**Saline**	23	26
	Saline	23 vs 26	P=0.000598	t=4.751
	HLS	23 vs 26	ns	x
**Preference**	**Saline vs HLS**	**HLS**	6	22
6vs22	ns	6		q=4.849
	ns	22	P=0.0416	
**22vs27**	ns	**27**	22	27
ns		22		x
ns		27	ns	
**6vs27**			6	27
ns		6		x
ns		27	ns	
**Latency 6vs22**	**Saline vs HLS**	**HLS**	6	22
Group F=8.544 p<0.01	ns	6		q=3.563
	t=2.825 P=0.0182	22	P=0.0393	
**22vs27**	ns	**27**	22	27
ns		22		x
ns		27	ns	
**6vs27**			6	27
ns		6		x
ns		27	ns	
**Crossing 6vs22**	**Saline vs HLS**	**HLS**	6	22
	ns	6		q=3.916
	ns	22	0.0853	
**22vs27**	ns	**27**	22	27
ns		22		x
ns		27	ns	
**6vs27**			6	27
ns		6		x
ns		27	ns	

Statistic table to figure 1.

Statistic to Figure 2

Two Way ANOVA	Saline vs ChABC		Saline			ChABC		
**FIB-SEM**			37	16	37post	37	16	37post
		37		q=5.606	q=5.485		q=12.3	q=5.528
Temp F(1.839,239.1)= 39.27, p<0.0001	ns	16	0.0005		q=9.855	0.0001		q=4.97
Interaction F(2,260)= 19.4, p<0.0001	t= 3.821, p<0.001	37post	0.0007	0.0001		0.0006	0.0023	
**Vgat/Geph**			37	16	37post	37	16	37post
	ns	37		q=5.384	x		q=3.474	x
Temp. F (1.951,29.69)=11.50 p=0.0002	ns	16	0.0208		q=4.252	0.098		q=5.642
	ns	37post	ns	0.0358		ns	0.0127	
**Vglut1/PSD95**			37	16	37post	37	16	37post
Group F (1,45) =9.128 p=0.0041	ns	37		q=7.137	q=3.881		q=11.84	q=4.408
Temp. F (1.747,39.32)=35.71 p<0.0001	ns	16	0.0037		q=5.848	0.0002		q=9.33
Interaction F (2,45) = 14.61 p<0.0001	q=4.709 p<0.01	37post	ns	0.0106		0.0396	0.0008	

One Way ANOVA			ChABC		One Way ANOVA			ChABC		One Way ANOVA
**Vgat/Geph**	F (3.28) =27.24 p<0.0001				**Vglut1/PSD95**	F (3,27) =6.882 p=0.0014				
	Saline	HLS	ChABC	ChABC +HLS		Saline	HLS	ChABC	ChABC +HLS	
Saline		q=4.112	q=5.760	q=6.855	Saline		q=4.702	x	x	
HLS	0.0336		q=9.881	q=11.17	HLS	0.0128		q=6.119	q=3.9	
ChABC	0.0019	0.0001		x	ChABC	ns	0.001		x	
ChABC + HLS	0.0002	0.0001	ns		ChABC + HLS	ns	0.0477	ns		

Two way ANOVA	FloxP vs AcanKO		FloxP			AcanKO		
**Vgat/Geph**			37	16	37post	37	16	37post
	ns	37		x	x		q=10.83	x
Temp. F(1.817,39.07)=17.27 p<0.0001	q=3.124 p=0.0243	16	ns		x	0.0006		q=8.382
Interaction F (2,43) =5.802 p<0.01	ns	37post	ns	ns		ns	0.0025	
**Vglut1/PSD95**			37	16	37post	37	16	37post
	ns	37		q=4.37	x		q=6.588	x
Temp. F (1.727,23.31) =15.83 p<0.0001	ns	16	0.0487		q=3.694	0.0057		q=6.087
	ns	37post	ns	0.079		ns	0.0087	

One Way ANOVA				AcanKO	One Way ANOVA				AcanKO
**Vgat/Geph**	F (3,27)= 12.173 p<0.0001				**Vglut1/PSD95**	F (3,26)= 8.051 p<0.001			
	FloxP	FloxP + HLS	AcanKO	AcanKO +HLS		FloxP	FloxP + HLS	AcanKO	AcanKO +HLS
FloxP		q=6.069	x	x	FloxP		x	x	q=4.050
FloxP + HLS	0.0011		q=8.091	q=5.646	FloxP + HLS	ns		q=5,327	q=6191
AcanKO	ns	0.0001		x	AcanKO	ns	0.0045		x
AcanKO +HLS	ns	0.0024	ns		AcanKO +HLS	0.0384	0.0009	ns	

Statistic table to figure 2.

Statistic to Figure 3									
**Two Way ANOVA**		**Saline vs ChABC**		**Saline**			**ChABC**		
	**PSD95**			37	16	37post	37	16	37post
Group	ns	ns	37		x	q=5.036		x	q=4.784
Temp.	F (1.435,24.39)=4.703 p=0.0238	ns	16	ns		x	ns		x
Interaction	ns	ns	37post	0.0501	ns		0.0436	ns	
	**SNAP25**			37	16	37post	37	16	37post
Group	F(1,4)=32.85 p=0.0046	ns	37		q=6.111	x		x	q=14.34
Temp.	F=63.21 p=0.001	q= 7.62 p<0.05	16	0.0891		q=12.92	ns		q=16.21
Interaction	F=21.54 p=0.006	q= 10.33 p<0.01	37post	ns	0.0214		0.0175	0.0137	
	**VGLUT1**			37	16	37post	37	16	37post
Group	ns	ns	37		x	x		q=5.13	x
Temp.	F (1.984,27.78)=11.50 p=0.0002	ns	16	ns		x	0.0461		q=8.954
Interaction	ns	ns	37post	ns	ns		ns	0.007	
	**VGAT**			37	16	37post	37	16	37post
Group	ns	ns	37		x	x		x	x
Temp.	Ns	ns	16	0.0181		x	ns		x
Interaction	ns	ns	37post	ns	ns		ns	ns	

Statistic table to figure 3.

Statistic to Figure 4											
**Delta - memory**	ANOVA F (3,48)=2.493 p=0.0759					ns				**Delta - memory**	
5 vs 23	23	Saline	HLS	ChABC	ChABC +HLS	FloxP	FloxP + HLS	AcanKO	AcanKO + HLS	5 vs 23	5 vs 23
	Saline		q=3.793	x	x		q=4.433	x	x	FloxP	ns
t=3.424 p=0.0051	HLS	0.0421		q=4.440	x	0.0142		x	x	FloxP + HLS	ns
	ChABC	ns	0.0119		q=3.708	ns	ns		x	AcanKO	ns
t=2.838 p=0.0263	ChABC + HLS	ns	ns	0.0491		ns	ns	ns		AcanKO + HLS	ns
Re-learning	Two way RM ANOVA group F(3,48)=4.351 p=0.009					Two way RM ANOVA group F=8.422 p<0.001				Re-learning	
**Group- Post hoc test**	23	Saline	HLS	ChABC	ChABC +HLS	FloxP	FloxP + HLS	AcanKO	AcanKO + HLS	23	**Group- Post hoc test**
	Saline		x	x	x		q=5.849	x	x	FloxP	
	HLS	ns		x	x	0.0173		x	x	FloxP + HLS	
	ChABC	ns	ns		x	ns	ns		x	AcanKO	
	ChABC + HLS	ns	ns	ns		ns	ns	ns		AcanKO + HLS	
	24	Saline	HLS	ChABC	ChABC +HLS	FloxP	FloxP + HLS	AcanKO	AcanKO + HLS	24	
	Saline		x	x	x		q=4.442	x	x	FloxP	
	HLS	ns		q=3.756	x	0.0496		q=4.437	x	FloxP + HLS	
	ChABC	ns	0.0685		x	ns	0.0469		x	AcanKO	
	ChABC + HLS	ns	ns	ns		ns	ns	ns		AcanKO + HLS	
23 vs 26	25	Saline	HLS	ChABC	ChABC +HLS	FloxP	FloxP + HLS	AcanKO	AcanKO + HLS	25	23 vs 26
t=4.751 p=0.000598	Saline		x	x	x		q=4.375	x	x	FloxP	t=4.219 p=0.003942
ns	HLS	ns		x	x	0.0577		x	x	FloxP + HLS	ns
ns	ChABC	ns	ns		x	ns	ns		x	AcanKO	ns
t=2.885 p=0.01369	ChABC + HLS	ns	ns	ns		ns	ns	ns		AcanKO + HLS	ns
Training 1 vs 5	26	Saline	HLS	ChABC	ChABC +HLS	FloxP	FloxP + HLS	AcanKO	AcanKO + HLS	26	Training 1 vs 5
q=7.520 p<0.0001	Saline		q=4.382	x	x		q=4.409	x	x	FloxP	q=7.330 p<0.0001
q=9.642 p<0.0001	HLS	0.0345		q=4.640	x	0.0837		x	x	FloxP + HLS	q=6.103 p=0.0003
q=7.725 p<0.0001	ChABC	ns	0.0233		x	ns	ns		x	AcanKO	q=4.845 p=0.0077
q=8.780 p<0.0001	ChABC + HLS	ns	ns	ns		ns	ns	ns		AcanKO + HLS	q=7.572 p<0.0001

Statistic table to figure 4.

Statistic to Figure 5											
Two way ANOVA	ChABC					AGGKO					Two way ANOVA
Preference		ns				F (3,27)= 5.282 p=0.0054				Preference	
6 vs 22	day 6	Saline	HLS	ChABC	ChABC +HLS	FloxP	FloxP + HLS	AcanKO	AcanKO + HLS	day 6	6 vs 22
ns	Saline		x	x	x		x	x	x	FloxP	ns
q=4,565 p<0.002	HLS	ns		x	x	ns		x	x	FloxP + HLS	q=4.789 p<0.05
ns	ChABC	ns	ns		x	ns	ns		x	AcanKO	ns
ns	ChABC + HLS	ns	ns	ns		ns	ns	ns		AcanKO + HLS	ns
22 vs 27	day22	Saline	HLS	ChABC	ChABC +HLS	FloxP	FloxP + HLS	AcanKO	AcanKO + HLS	day22	22 vs 27
ns	Saline		x	x	x		x	x	x	FloxP	ns
q=3.644 p=0.0324	HLS	ns		x	x	ns		q=3.846	q=4.499	FloxP + HLS	ns
q=4.453 p=0.0136	ChABC	ns	ns		x	ns	0.0392		x	AcanKO	ns
ns	ChABC + HLS	ns	ns	ns		ns	0.011	ns		AcanKO + HLS	ns
6 vs 27	day27	Saline	HLS	ChABC	ChABC +HLS	FloxP	FloxP + HLS	AcanKO	AcanKO + HLS	day27	6 vs 27
ns	Saline		x	x	x		q=3.911	x	x	FloxP	ns
ns	HLS	ns		x	x	0.0348		x	q=3.619	FloxP + HLS	ns
q= 6.258 p=0.0087	ChABC	ns	ns		x	ns	ns		x	AcanKO	s
ns	ChABC + HLS	ns	ns	ns		ns	0.0586	ns		AcanKO + HLS	q=3.977 p<0.05
Latency		F(3,48)=4.269 p=0.0094				F(3,27)=5.426 p=0.0033				Latency	
6 vs 22	day 6	Saline	HLS	ChABC	ChABC +HLS	FloxP	FloxP + HLS	AcanKO	AcanKO + HLS	day 6	6 vs 22
ns	Saline		x	x	x		x	x	x	FloxP	ns
q=3.699 p=0.0227	HLS	ns		x	x	ns		x	x	FloxP + HLS	q=3.814 p=0.0249
ns	ChABC	ns	ns		x	ns	ns		x	AcanKO	ns
ns	ChABC + HLS	ns	ns	ns		ns	ns	ns		AcanKO + HLS	ns
22 vs 27	day22	Saline	HLS	ChABC	ChABC +HLS	FloxP	FloxP + HLS	AcanKO	AcanKO + HLS	day22	22 vs 27
ns	Saline		q=4.047	x	x		q=4.356	x	x	FloxP	ns
ns	HLS	0.022		q=4.012	x	0.0147		x	x	FloxP + HLS	ns
ns	ChABC	ns	0.03		x	ns	ns		x	AcanKO	ns
ns	ChABC + HLS	ns	ns	ns		ns	ns	ns		AcanKO + HLS	ns
6 vs 27	day27	Saline	HLS	ChABC	ChABC +HLS	FloxP	FloxP + HLS	AcanKO	AcanKO + HLS	day27	6 vs 27
ns	Saline		x	x	x		q=4.42	x	x	FloxP	ns
ns	HLS	ns		x	x	0.0129		x	q=4.703	FloxP + HLS	q=3.904 p=0.0211
ns	ChABC	ns	ns		x	ns	ns		x	AcanKO	ns
ns	ChABC + HLS	ns	ns	ns		ns	0.0071	ns		AcanKO + HLS	ns

Statistic table to figure 5.

Statistic to Figure S3 S4								
Two Way ANOVA	Saline vs ChABC		Saline			ChABC		
Bassoon			37	16	37post	37	16	37post
Group F (1,53)= 22.56, p<0.0001	q=3.848 p=0.0012	37		q=4.16	x		q=7.65	x
Temp. F(1.82,70.06) =24.25, p<0.0001	ns	16	0.0235		q=5.71	0.0001		q=9.9463
Interaction F (2,77) =6.907, p=0.0017	q=5.018 p<0.0001	37post	ns	0.0029		ns	0.0001	
VGAT			37	16	37post	37	16	37post
Group F (1,63)=5.205 p=0.0259	ns	37		q=21.22	q=5.177		q=6.113	x
Temp. F(1.681,80.68)=85.39 p<0.0001	ns	16	0.0001		q=10.48	0.0016		q=13.6
Interaction F(2,96)= 6.373 p=0.0025	ns	37post	0.0033	0.0001		ns	0.0001	

One Way ANOVA			ChABC	
Bassoon	F (3,109) =12.07 p<0.0001			
	Saline	HLS	ChABC	ChABC +HLS
Saline		x	q=6.048	x
HLS	ns		q=8.255	q=3.605
ChABC	0.0002	0.0001		q= 4.648
ChABC + HLS	ns	0.058	0.0074	

One Way ANOVA			ChABC	
VGAT	F (3,112)=5.909 p<0.001			
	Saline	HLS	ChABC	ChABC +HLS
Saline		q= 4.144	q= 4.945	q= 5.147
HLS	0.0211		x	x
ChABC	0.0037	ns		x
ChABC + HLS	0.0023	ns	ns	

Two way ANOVA	FloxP vs AcanKO		FloxP			AcanKO		
Bassoon			37	16	37post	37	16	37post
Group F (1,138)=7.226 p<0.0037	ns	37		q=9.488	x		q=6.835	x
Temp. F (1.51, 104.2)=51.375 p<0.0001	ns	16	0.0001		q=12.11	0.0014		q=14.87
Interaction ns	ns	37post	ns	0.0001		ns	0.0001	
VGAT			37	16	37post	37	16	37post
Group F (1.55) =29.39 p<0.0001	ns	37		q=7.012	q=4.8		q=8.475	x
Temp. F (1.745,68.92) =42.54 p<0.0001	t=5.701 p<0.0001	16	0.0002		q=10.68	0.0002		q=14.31
Interaction F (2,79) =4.965 p<0.0093	t=5.96 p<0.0001	37post	0.0055	0.0001		ns	0.0001	

One Way ANOVA				AcanKO
Bassoon	F (3,86) = 5.905 p=0.001			
	FloxP	FloxP + HLS	AcanKO	AcanKO +HLS
FloxP		q=4.077	x	x
FloxP + HLS	0.0252		q= 5.636	q=4.507
AcanKO	ns	0.0008		x
AcanKO +HLS	ns	0.0106	ns	

One Way ANOVA				AcanKO
VGAT	F (3,111)= 20 p<0.0001			
	FloxP	FloxP + HLS	AcanKO	AcanKO +HLS
FloxP		q=7.344	x	q=10.08
FloxP + HLS	0.0001		q=4.257	x
AcanKO	ns	0.0168		q= 6.828
AcanKO +HLS	0.0001	ns	0.0001	

Statistic table to S3,4

Statistic to Figure S5	One Way ANOVA			
**PSD95**	ns			
	Saline	HLS	ChABC	ChABC +HLS
Saline		x	x	x
HLS	ns		x	x
ChABC	ns	ns		x
ChABC + HLS	ns	ns	ns	
**SNAP25**	F (3,30)=4.503 p=0.0101			
	Saline	HLS	ChABC	ChABC +HLS
Saline		x	q=4.367	q=3.531
HLS	ns		q=3.694	x
ChABC	0.0212	0.0633		x
ChABC + HLS	0.0809	ns	ns	
**VGLUT1**	F (3,30)=6.701 p=0.0014			
	Saline	HLS	ChABC	ChABC +HLS
Saline		x	q=6.194	x
HLS	ns		x	x
ChABC	0.0007	ns		q=4.15
ChABC + HLS	ns	ns	0.0306	
**VGAT**	ns			
	Saline	HLS	ChABC	ChABC +HLS
Saline		x	x	x
HLS	ns		x	x
ChABC	ns	ns		x
ChABC + HLS	ns	ns	ns	

Statistic table to figure S5

**Statistic to Figure S6**				Saline			ChABC		
**GAD 65/67 - acute**				37	16	37post	37	16	37post
Group	F(1,19)=5.76 p<0.05	ns	37		x	q=3.575		x	x
Temp.	ns	ns	16	ns		x	ns		x
Interaction	ns	q= 2.777 p=0.0531	37post	0.0887	ns		ns	ns	

**GAD65/67 - long term**	F(3,30)=2.754 p=0.0598				
	Saline	HLS	ChABC	ChABC +HLS	
Saline		q=3.690	x	x	Saline
HLS	0.0637		x	x	HLS
ChABC	ns	ns		x	ChABC
ChABC + HLS	ns	ns	ns		ChABC + HLS

Statistic table to figure S6

Statistic to Figure S7											
**Crossing**		ns				$F(3,27)=5.563$ $p=0.0035$				**Crossing**	
6 vs 22	day6	Saline	HLS	ChABC	ChABC +HLS	FloxP	FloxP + HLS	AcanKO	AcanKO + HLS	day6	6 vs 22
ns	Saline		x	x	x		x	x	x	FloxP	ns
ns	HLS	ns		x	x	ns		x	x	FloxP + HLS	q=4.00 p=0.0674
q=3.916p=0.085	ChABC	ns	ns		x	ns	ns		x	AcanKO	ns
ns	ChABC + HLS	ns	ns	ns		ns	ns	ns		AcanKO + HLS	ns
22 vs 27	day22	Saline	HLS	ChABC	ChABC +HLS	FloxP	FloxP + HLS	AcanKO	AcanKO + HLS	day22	22 vs 27
ns	Saline		x	x	x		q=3.517	x	x	FloxP	ns
ns	HLS	ns		x	x	0.0696		x	x	FloxP + HLS	ns
q=3.326 p=0.084	ChABC	ns	ns		x	ns	ns		x	AcanKO	ns
ns	ChABC + HLS	ns	ns	ns		ns	ns	ns		AcanKO + HLS	ns
6 vs 27	day27	Saline	HLS	ChABC	ChABC +HLS	FloxP	FloxP + HLS	AcanKO	AcanKO + HLS	day27	6 vs 27
ns	Saline		x	x	x		q=5.77	x	x	FloxP	ns
ns	HLS	ns		q=4.588	x	0.006		q=4.269	q=3.828	FloxP + HLS	q=7.473 p=0.0045
ns	ChABC	ns	0.015		x	ns	0.0175		x	AcanKO	ns
ns	ChABC + HLS	ns	ns	ns		ns	0.0405	ns		AcanKO + HLS	ns
**Quadrant**		ns				$F(3,27)= 3.708$ $p=0.0335$				**Quadrant**	
6 vs 22	day27	Saline	HLS	ChABC	ChABC +HLS	FloxP	FloxP + HLS	AcanKO	AcanKO + HLS	day27	6 vs 22
ns	Saline		x	x	x		x	x	x	FloxP	ns
q=2.749 p=0.069	HLS	ns		x	x	ns		x	x	FloxP + HLS	q=4.762 p=0.0348
q=2.99 p=0.05	ChABC	ns	ns		x	ns	ns		x	AcanKO	ns
q=6.004 p<0.001	ChABC + HLS	ns	ns	ns		ns	ns	ns		AcanKO + HLS	ns
22 vs 27	day27	Saline	HLS	ChABC	ChABC +HLS	FloxP	FloxP + HLS	AcanKO	AcanKO + HLS	day27	22 vs 27
ns	Saline		x	x	x		x	x	x	FloxP	ns
q=2.611 p=0.083	HLS	ns		x	x	ns		x	x	FloxP + HLS	q=3.703 p=0.0878
q=2.687 p=0.075	ChABC	ns	ns		x	ns	ns		x	AcanKO	ns
q=2.959 p=0.052	ChABC + HLS	ns	ns	ns		ns	ns	ns		AcanKO + HLS	ns
6 vs 27	day27	Saline	HLS	ChABC	ChABC +HLS	FloxP	FloxP + HLS	AcanKO	AcanKO + HLS	day27	6 vs 27
ns	Saline		x	x	x		q=3.976	x	x	FloxP	ns
ns	HLS	ns		x	x	0.0309		q=3.671	x	FloxP + HLS	ns
ns	ChABC	ns	ns		q=3.585	ns	0.0535		x	AcanKO	ns
ns	ChABC + HLS	ns	ns	0.082		ns	ns	ns		AcanKO + HLS	ns

Statistic table to figure S7

Statistic to figure S9					
ns				Delta - memory	
FloxP	FloxP + HLS	AcanKOgl	AcanKOgl + HLS	5 vs 23	5 vs 23
	x	x	x	FloxP	ns
ns		x	x	FloxP + HLS	ns
ns	ns		x	AcanKOgl	ns
ns	ns	ns		AcanKO gl+ HLS	ns
Two way RM ANOVA group F=11.087 p<0.001				Re-learning	
FloxP	FloxP + HLS	AcanKOgl	AcanKOgl + HLS	23	**Group- Post hoc test**
	x	x	q=4.135	FloxP	
ns		x	x	FloxP + HLS	
ns	ns		q=4.244	AcanKOgl	
0.0219	ns	0.0176		AcanKO gl+ HLS	
FloxP	FloxP + HLS	AcanKOgl	AcanKOgl + HLS	24	
	q=3.482	x	q=3.687	FloxP	
0.0722		x	x	FloxP + HLS	
ns	ns		x	AcanKOgl	
0.0507	ns	ns		AcanKO gl+ HLS	
FloxP	FloxP + HLS	AcanKOgl	AcanKOgl + HLS	25	23 vs 26
	x	x	x	FloxP	t=2.92 p=0.065523
ns		x	x	FloxP + HLS	t=2.458 p=0.085313
ns	ns		x	AcanKOgl	ns
ns	ns	ns		AcanKO gl+ HLS	t=3.762 p=0.03696
FloxP	FloxP + HLS	AcanKOgl	AcanKOgl + HLS	26	Training 1 vs 5
	x	x	x	FloxP	q=7.085 p<0.0001
ns		x	x	FloxP + HLS	q=5.959 p=0.0013
ns	ns		x	AcanKOgl	q=5.987 p=0.0005
ns	ns	ns		AcanKO gl+ HLS	q=5.895 p=0.0006

Statistic table to figure S9

statistic to Figure S10											
AGGKO Global					Two way ANOVA	AGGKO Global					Two way ANOVA
ns				Preference		ns				Crossing	
FloxP	FloxP + HLS	AcanKOgl	AcanKOgl + HLS	day 6	6 vs 22	FloxP	FloxP + HLS	AcanKOgl	AcanKOgl + HLS	day6	6 vs 22
	x	x	x	FloxP	ns		x	x	x	FloxP	ns
ns		x	x	FloxP + HLS	q=4.046 p=0.0561	ns		x	x	FloxP + HLS	q=3.901 p=0.0645
ns	ns		x	AcanKOgl	ns	ns	ns		x	AcanKOgl	ns
ns	ns	ns		AcanKOgl + HLS	ns	ns	ns	ns		AcanKOgl + HLS	ns
FloxP	FloxP + HLS	AcanKOgl	AcanKOgl + HLS	day22	22 vs 27	FloxP	FloxP + HLS	AcanKOgl	AcanKOgl + HLS	day22	22 vs 27
	x	x	x	FloxP	ns		x	x	x	FloxP	ns
ns		x	x	FloxP + HLS	q=6.145 p=0.0082	ns		x	x	FloxP + HLS	q=4.068 p= 0.0549
ns	ns		x	AcanKOgl	ns	ns	ns		x	AcanKOgl	ns
ns	ns	ns		AcanKOgl + HLS	q=4.688 p=0.037	ns	ns	ns		AcanKOgl + HLS	ns
FloxP	FloxP + HLS	AcanKOgl	AcanKOgl + HLS	day27	6 vs 27	FloxP	FloxP + HLS	AcanKOgl	AcanKOgl + HLS	day27	6vs 27
	x	x	x	FloxP	ns		x	x	x	FloxP	ns
ns		x	x	FloxP + HLS	ns	ns		q=3.931	x	FloxP + HLS	ns
ns	ns		x	AcanKOgl	ns	ns	0.0717		x	AcanKOgl	ns
ns	ns	ns		AcanKOgl + HLS	ns	ns	ns	ns		AcanKOgl + HLS	ns
F=5.426 p<0.01				Latency		ns				Quadrant	
FloxP	FloxP + HLS	AcanKOgl	AcanKOgl + HLS	day 6	6 vs 22	FloxP	FloxP + HLS	AcanKOgl	AcanKOgl + HLS	day27	6 vs 22
	x	x	x	FloxP	ns		x	x	x	FloxP	ns
ns		q=5.153	x	FloxP + HLS	q=4.593 p=0.0332	ns		x	x	FloxP + HLS	ns
ns	0.0155		x	AcanKOgl	ns	ns	ns		x	AcanKOgl	ns
ns	ns	ns		AcanKOgl + HLS	ns	ns	ns	ns		AcanKOgl + HLS	ns
FloxP	FloxP + HLS	AcanKOgl	AcanKOgl + HLS	day22	22 vs 27	FloxP	FloxP + HLS	AcanKOgl	AcanKOgl + HLS	day27	22 vs 27
	ns	x	x	FloxP	ns		x	x	x	FloxP	ns
ns		q=4.411	x	FloxP + HLS	q=4.155 p=0.0505	ns		x	x	FloxP + HLS	q=5.425 p=0.0154
ns	0.0399		x	AcanKOgl	ns	ns	ns		x	AcanKOgl	ns
ns	ns	ns		AcanKOgl + HLS	ns	ns	ns	ns		AcanKOgl + HLS	ns
FloxP	FloxP + HLS	AcanKOgl	AcanKOgl + HLS	day27	6 vs 27	FloxP	FloxP + HLS	AcanKOgl	AcanKOgl + HLS	day27	6 vs 27
	x	x	x	FloxP	ns		x	x	x	FloxP	ns
ns		x	x	FloxP + HLS	ns	ns		x	x	FloxP + HLS	q=7.804 p=0.0022
ns	ns		x	AcanKOgl	q=1.72 p=0.0737	ns	ns		x	AcanKOgl	ns
ns	ns	ns		AcanKOgl + HLS	ns	ns	ns	ns		AcanKOgl + HLS	ns

Statistic table to figure S10
